# Transcriptional profile of genes involved in the production of terpenes and glyceollins in response to biotic stresses in soybean

**DOI:** 10.1590/1678-4685-GMB-2019-0388

**Published:** 2020-11-06

**Authors:** Talitta Regina Parmezan, Salvador Lima Brito, Kenia de Carvalho, Moisés de Aquino, Michael Birkett, John Pickett, Estela de Oliveira Nunes, Ricardo Vilela Abdelnor, Clara Beatriz Hoffmann Campo, Francismar Corrêa Marcelino-Guimarães

**Affiliations:** ^1^ Universidade Estadual de Londrina, Departamento de Bioquímica e Biotecnologia, Londrina, PR, Brazil.; ^2^ GDM Genética do Brasil, Londrina, PR, Brazil.; ^3^ Universidade Estadual de Londrina, Departamento de Genética e Biologia Molecular, Londrina, PR, Brazil.; ^4^ Empresa Brasileira de Pesquisa Agropecuária-EMBRAPA Soja, Londrina, PR, Brazil.; ^5^ Rothamsted Research, Biointeractions and Crop Protection Department, Harpenden, UK.; ^6^ Cardiff University, School of Chemistry, Wales, UK.

**Keywords:** Rust, Glycine max, terpenes, glyceollin

## Abstract

Terpenes produced by plants comprise a diverse range of secondary metabolites, including volatile organic compounds (VOCs). Terpene VOC production may be altered after damage or by biological stimuli such as bacterial, fungal and insects, and subsequent triggering of plant defense responses. These VOCs originate in plants from two independent pathways: the mevalonate and the methylerythritol phosphate pathways, which utilize dimethylallyl and isopentenyl diphosphates to form the terpenoidal precursors. *Phakopsora pachyrhizi* fungi causes Asian soybean rust, limiting soybean production and resulting in losses of up to 80% if no control strategies are applied. By using a transcriptome datasets, we investigated the regulation of genes of the mevalonate pathway under different biotic stresses. We studied the impact of *P. pachyrhizi* infection *in vivo* expression profile of genes involved in terpenoid and glyceollin biosynthesis in genotypes harboring different resistance genes (*Rpp*), and across the infection cycle. In addition, we used UPLC and UPGC analysis to evaluate glyceollin and VOC production, respectively, to identify metabolites associated with soybean responses to pathogen infection. The regulation of soybean genes involved in terpene production was influenced by genotypes, depending on the *Rpp* gene*,* while glyceollin was induced in all genotypes. Furthermore, a sesquiterpene was identified as a potential marker associated with rust symptoms on soybean.

## Introduction

Terpenes constitute the largest and most diversified class of secondary metabolites, including some volatile organic compounds (VOCs) formed from the combination of two or more isoprenyl units, with the molecular formula (C_5_H_8_O_2_)_n_. These compounds constitute some signaling molecules in plants and are commonly produced in response to bacteria, fungi and insects infections (Mendgen *et al.*, 2006; Huang *et al.*, 2012; Pickett *et al.*, 2012; Tamiru *et al.*, 2012). Under infection, plants trigger an arsenal of chemical and physical reactions, in responses to stresses and this induction is directly related with type of injury (Piesik *et al.*, 2010). In addition, these plants signaling molecules may have negative effects on fungal infection processes (Mendgen *et al.*, 2006). As signaling molecules, some terpenes can operate by stimulating plant defense mechanisms, including those of its intact systemic parts, or even by stimulating recognition in nearby plants (Baldwin *et al.*, 2006; Heil and Silva Bueno, 2007), and could be useful as markers of the plant resistance or the infection process, especially if detected at initial steps of infection.

Biosynthesis of plant terpenes occurs in two independent pathways: the methylerythritol phosphate (MEP) pathway, located in the plastid, and the mevalonate (MVA) pathway, located in the cytosol, although with some emerging exceptions and crossovers. The MVA pathway is responsible for the production of the precursors of various terpenes and specifically the sesquiterpenes from dimethylallyl diphosphate (DMAPP) and isopentenyl diphosphate (IPP), while the MEP pathway provides these precursors for the formation of hemiterpenes, monoterpenes, diterpenes and isoprenes, which are precursors for the prenylation step towards a variety of phytoalexins, such as glyceollins (Akashi *et al.*, 2009).

Glyceollins are prenylated pterocarpans, regarded as the main of soybean phytoalexins and are important in the defense against phytopathogens (Gouvea *et al.*, 2011). Glyceollins have antioxidant, antimicrobial, antinematode, and antifungal actions. The two main precursors in glyceollin production are daidzein and DMAPP, derived from phenylpropanoids and the MEP pathway, respectively (Akashi *et al.*, 2009). According to the authors, DMAPP provides the isoprenyl unit for the enzyme prenylase 4-dimethylallyl transferase (G4DT, E.C. 2.5.1.36), which catalyzes the addition of DMAPP to carbon C_2_ or C_4_ of the pterocarpan skeleton (glycinol). The contribution of the MVA pathway to glyceollin production occurs at the step catalyzed by isopentenyl diphosphate Δ-isomerase, which converts IPP into DMAPP, which can be transported from the cytoplasm to the chloroplast and elongate the pterocarpan skeleton formed from daidzein (Lygin *et al.*, 2009).

Asian soybean rust (ASR) is a disease caused by the obligate biotrophic fungus *Phakopsora pachyrhizi* (Sydow and P. Syd). Damage caused by rust in Brazil varies from 10% to 80% (Sinclair and Hartman, 1999; Yorinori *et al.*, 2005), depending on the developmental stage of the plant and the degree of severity of the infection. Seven main genes conferring resistance to ASR (R genes) have been identified: *Rpp*1, *Rpp*2, *Rpp*3, *Rpp*4, *Rpp*5, *Rpp*6 and *Rpp*7, and genetically mapped in the soybean genome (Bromfield and Hartwig, 1980; Mclean and Byth, 1980; Hartwig and Bromfield, 1983; Hartwig, 1986; Hyten *et al.*, 2007; Monteros *et al.*, 2007; Garcia *et al.*, 2008; Silva *et al.*, 2008; Chakraborty *et al.*, 2009; Kim *et al.*, 2012; Li *et al.*, 2012; Childs *et al.*, 2018). However, the development of soybean cultivars with broad and durable resistance against *P. pachyrhizi* is not achieved so far.

Recent studies have focused on identifying mechanisms of defense response to infection in soybean genotypes, by correlating transcriptomic, metabolomic and functional genomic approaches. These studies have shown that soybean accessions harboring different major *Rpp* genes can trigger resistance responses related to some metabolic pathways, and compounds formed by these pathways are involved in defense mechanisms (van de Mortel *et al.*, 2007; Choi *et al.*, 2008; Pandey *et al.*, 2011; Schneider *et al.*, 2011; Tremblay *et al.*, 2011; Morales *et al.*, 2013). Studies on the transcriptomic response of soybean to *P. pachyrhizi* infection identified differentially expressed genes from the MVA pathway, such as those encoding the enzymes diphosphomevalonate decarboxylase (Choi *et al.*, 2008), mevalonate kinase (Tremblay *et al.*, 2010), acetyl-CoA acetyltransferase and phosphomevalonate kinase (Tremblay *et al.*, 2011), geranyl diphosphate synthase and hydroxymethylglutaryl-CoA synthase (Schneider *et al.*, 2011). According to Mendgen *et al.* (2006), *P. pachyrhizi* development in soybean (cv Erin) leaves was dependent on the mixture of three compounds (nonanal, decanal, and hexenyl acetate). This blend was able to promote development of the pathogen 24 hours after inoculation (hai). However, the terpenoid farnesyl acetate, when separately evaluated, negatively regulated haustorial development, by suppressing differentiation of the fungal cells, and reducing 98% of colony development. However, it is unknown whether this phenotype was caused by induction of soybean defense mechanisms by VOC or whether the compound itself has an antifungal effect (Mendgen *et al.*, 2006).

Studies in soybeans have demonstrated that induction of glyceollin synthesis is a defense response of different types of stress (Mazaro *et al.*, 2008; Akashi *et al.*, 2009; Gouvea *et al.*, 2011; Silva, 2013; Liu *et al.*, 2014). Akashi *et al.* (2009) reported an up-regulation of glyceollin production in soybean in response to *P. pachyrhizi* after one week of infection, associated with traditional pathway (MEP), involving the precursor daidzein and dimethylallyl diphosphate (DMAPP), as well as the action of the G4DT enzyme. Naoumkina *et al.* (2007) and Farag *et al.* (2008) suggested that the intermediate of phytoalexins, the isoflavones, as daidzein are stored as glycosides (daidzin), facilitating the rapid synthesis of the glyceollins following a pathogenic attack to legume plants. Lygin *et al.* (2009) also reported glyceollin accumulation when studying differences in phenolic metabolism during a three-week soybean-*P. pachyrhizi* interaction. Additionally, they observed that this phenomenon was mainly present in genotypes containing resistance genes.

In the present study, genes involved in the cytoplasmic pathway for IPP production and genes encoding different prenylases involved in the production of mono-, di-, and sesquiterpenes and glyceollin synthesis were evaluated regarding their response to different soybean pathogens by *in silico* analysis of transcriptome databases. Using RT-qPCR, the expression levels of these genes were also evaluated in response to *P. pachyrhizi* infection*,* using soybean genotypes containing different *Rpp* genes. These genotypes were also evaluated for glyceollin accumulation during the infection cycle. We found that while glyceollin is a general metabolite activated in response to pathogen infection on soybean, including by *P. pachyrhizi*, and the production of different classes of terpenoids was differentially regulated depending on the *Rpp* gene present in the soybean accessions. Additionally, VOC detection was carried out on the resistant source of Rpp2 and the susceptible genotype Embrapa 48. Variations of the VOC (*E,E*)-α-farnesene were detected in the initial hours after *P. pachyrhizi* inoculation, either in compatible (genotype Embrapa 48) or incompatible (PI 230970) interactions of the soybean with rust. The levels of this VOC were inversely proportional to the plant symptoms; namely, the more evident the infection success was, the lower the VOC production, validating the expression profile observed in these soybean accessions and revealing it as a potential marker associated with of rust disease symptoms on soybean.

## Material and Methods

### *In sílico* analysis

The involvement of MVA pathway genes in soybean disease responses was assessed by examining their expression profiles during pathogen infection, which are available in transcriptome databases. The gene models previously reported as differentially expressed in *P. pachyrhizi* studies were used as selection criteria when different gene models were available for same enzyme annotation (van de Mortel *et al.*, 2007; Schneider *et al.*, 2011; Morales *et al.*, 2013).

The *in silico* analyses were conducted using data from nine microarray experiments performed with pathogen-infected soybeans, available at Genevestigator[1]. Data were available from experiments with the soybean pathogens *P. pachyrhizi*, and the oomycete *Phytophthora sojae* (Tyler, 2007; Gijzen *et al.*, 2009), the soybean aphid *Aphis glycines* (Studham and Macintosh, 2013), the nematode *Heterodera glycines* and the bacteria *Bradyrhizobium japonicum* (Zhou *et al.*, 2009; Libault *et al.*, 2010). The *in silico* expression analysis enabled evaluation of the transcriptional of MVA pathway genes during different biotic stresses in soybean. Unexpressed gene models in at least one of the experimental conditions were excluded from further analysis.

Seven genes encoding to the enzymes of the cytoplasmic MVA pathway acetyl-CoA acetyltransferase (E.C. 2.3.1.9), hydroxymethylglutaryl-CoA synthase (E.C. 2.3.3.10), mevalonate kinase (E.C. 2.7.1.36), phosphomevalonate kinase (E.C. 2.7.4.2), diphosphomevalonate decarboxylase (E.C. 4.1.1.33), and isopentenyl-diphosphate Δ-isomerase (E.C. 5.3.3.2) as well as four genes corresponding to the prenylases geranyl diphosphate synthase (E.C. 2.5.1.1), (2E, 6E)-farnesyl-diphosphate synthetase (E.C. 2.5.1.10), and geranyl-geranyl diphosphate synthase (E.C. 2.5.1.29) were include in this study. Likewise, genes encoding the terpene synthase (*E,E*) -α-farnesene synthase (GmTPS) (E.C. 4.2.3.46) and glyceollin synthase (E.C. 2.5.1.36) were also considered.

### *In vivo* analysis of genes involved in terpene and glyceollin production in soybean response to *P. pachyrhizi* infection

#### Experimental design

Soybean seeds of the cv Williams 82 (susceptible – W82) and the plant introductions (PIs) containing different *Rpp* genes to *P. pachyrhizi*, were obtained from the Embrapa Soja Germplasm Bank and soaked for 3 days in sand. Each genotype contains a specific previously characterized resistance (R) gene [PI 230970 (Rpp2), PI459025 (Rpp4) and PI200487 (Rpp5)]. The plants were grown in a greenhouse under controlled conditions of temperature (22 ? 1 °C), humidity (> 60%) and a photoperiod (12 h). After reaching V2 stage (Fehr and Caviness, 1981), the soybean plants were sprayed with the pathogen. The experiment was carried out by a completely randomized design with three replicates, each one composed by three plants. The Williams 82 (susceptible) and resistant PI genotypes were inoculated with pure isolated LUB55 (collected in Uberlandia – MG, Brazil, 2011), by manual spraying with a solution of water and 0.05% (v/v) Tween-20, and a final concentration of 5 × 10^4^ *P. pachyrhizi* urediniospores/ml. Samples were also mock-inoculated with the same solution lacking fungus (control plants). The second trifoliate from each plant was collected at 12, 24, 48, 72, 96 and 192 hours after inoculation (hai) and immediately transferred to liquid nitrogen and stored at −80 °C.

#### Plant RNA isolation

Each sample (100 mg per replicate) was separately ground with a pestle, mortar and liquid nitrogen. After, the samples were distributed into 1.5 mL microtubes and stored at −80 °C. Total RNA (~1 g) was isolated from *P. pachyrhizi*-infected or uninfected frozen leaves using an RNA extraction kit with TRIzol® reagent (Invitrogen, Carlsbad, CA, USA). Analysis to RNA quantifying and qualifying were performed with a Uniscience NanoDrop ND-1000 spectrophotometer (NanoDrop Technologies, Wilmington, DE, USA), at a wavelength of 230 nm, and via agarose gel electrophoresis, respectively. The RNA samples were treated with deoxyribonuclease I (Kit DNaseI, Invitrogen). The SuperScriptTM III Kit (Invitrogen) was used to synthesize cDNA from treated RNA according to the manufacturer's instructions, and, after, samples were stored at −20 °C. Validation of cDNA quality was performed using PCR with primers designed to anneal to two different exons of the soybean β-actin gene (forward, 5-CCCCTCAACCCAAAGG TCAACAG-3 and reverse, 5-GGAATCTCTCTGCCCCA ATTGTG-3), to avoid DNA contamination.

### RT-qPCR

**Figure 1:**
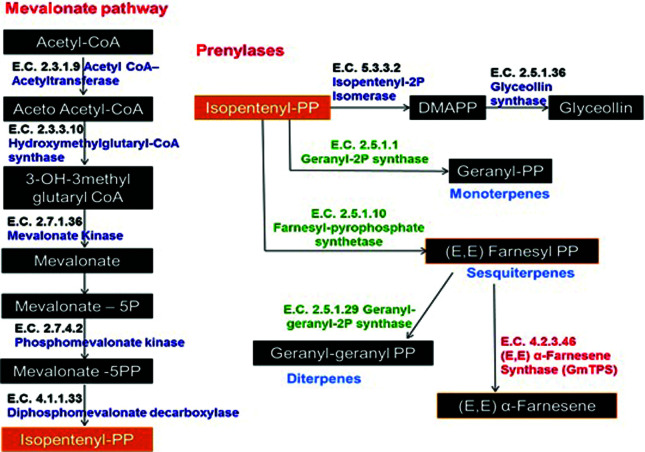
Fluxogram of mevalonate pathway genes evaluated by RT-qPCR and the different terpenes formed from isopentenyl diphosphate (IPP) through the action of prenylases, as well as terpene synthases (shown in red).

The expression profiles of the gene models involved in the biosynthesis of IPP (MVA pathway), terpenes and glyceollins during *P. pachyrhizi* infection were evaluated using RT-qPCR. The enzymes were selected using KEGG, and complete sequences of the gene models were obtained from Phytozome[2] and used to design primers. Specific primers to each of the 13 gene models were designed using the software Primer3Plus[3] and Vector NTI AdvanceTM (Invitrogen). The sequences of the primers are listed in Table S1. A schematic pathway composed of all the genes selected for the analysis is presented in Figure 1.

The cDNA samples were amplified with specific primers to each gene model at a final concentration of 0.1–0.5 μM using 1X Kit Platinum® SYBR® Green qPCR SuperMix UDG (Invitrogen–Life Technologies), according to the manufacturer's instructions. The β-actin gene was used as an endogenous control. The E = 10^(-1/slope)^ formula was employed to calculate the reaction efficiency and to adjust the final primer concentration and the calibration curve was established based on the Ct and the log of the cDNA dilutions. All amplification reactions were performed in triplicate using the 7900 Real Time System Thermocycler (Applied Biosystems), according to the manufacturer's instructions.[Table t1]

**Table 1 t1:** List of enzymes from the mevalonate pathway identified in KEGG and the corresponding gene models obtained from Phytozome V12.0.

Enzymes	ID Soybean	E. C.	Reference
Acetyl-CoA acetyltransferase	Glyma.17g005300	**2.3.1.9**	Tremblay *et al.,*2011
Hydroxymethylglutaryl-CoA synthase	Glyma.01g215500	**2.3.3.10**	Choi *et al.,*2008
Mevalonate kinase	Glyma.03g239000	**2.7.1.36**	Tremblay *et al.,*2010
Phosphomevalonate kinase	Glyma.06g127200	**2.7.4.2**	Tremblay *et al.,*2011
Diphosphomevalonate decarboxylase	Glyma.10g279800	**4.1.1.33**	Choi *et al.,*2008
	Glyma.20g109900		
Isopentenyl-diphosphate Δ-isomerase	Glyma.18g242300	**5.3.3.2**	-
Geranyl diphosphate synthase	Glyma.17g166000	**2.5.1.1**	-
(2E 6E)-Farnesyl-pyrophosphate synthetase	Glyma.15g121400	**2.5.1.10**	-
	Glyma.09g015600		
Geranyl-geranyl diphosphate synthase	Glyma.11g063900	**2.5.1.29**	Choi *et al.,*2008
Alpha-farnesene synthase (GmTPS)	Glyma.13g321100	**4.2.3.46**	Liu *et al.,*2014
Glyceollin synthase	Glyma.10g295300	**2.5.1.36**	-

After initial steps at 50 ºC for 2 min (UNG activity) and 95 ºC for 10 min (activation of the AmpliTaq Gold polymerase), a two-step program of 95 ºC for 15 s and 62 ºC for 1 min was run for 40 cycles. Dissociation curves were obtained to guarantee the absence of nonspecific amplification. The data were collected in the log phase, and the results were analyzed with the Sequence Detection program (Perkin Elmer, Waltham, MA, USA). The final relative quantification of each gene compared with the control was estimated considering the relative quantification (RQ) obtained in each biological replicate, as represented by each independent experiment, with three replicates each. Significant differences were defined based on estimates of the Relative Expression Software Tool (REST) version 2.0.7, with a significance level of 5%. Additionally, expression levels were converted to a log2-based colorimetric scale and represented as a heatmap using the software Cluster and Tree View (software copyright Stanford University 1998-99 and http://jtreeview.sourceforge.net, respectively).

### Glyceollin and (E,E) α-farnesene analysis

Aliquot portions of leaf samples from the RT-qPCR experiments were used for the analyses of glyceollin levels. Approximately 100 mg of fresh leaves were lyophilized and extracted with 5 mL of methanol (70% MeOH) using an AP59 vortex mixer (Phoenix Luterco®) until completely homogenized. After sonication (20 min) (ultrasonic cleaner LS-Logen), the samples were centrifuged (10,000 rpm for 10 min) and filtered through a Millipore filter (Ø = 0.22 μm).

The ultrafiltered samples were analyzed by liquid chromatography with data photo diode array detector (UHPLC-PDA) and mass detection (Xevo Q-Tof) (both devices manufactured by Waters®) at the Chemical Ecology Laboratory of Embrapa Soybean, Londrina, PR. Chromatographic reversed-phase conditions were performed as follows: a sample injection volume of 10 μL, a run time of 17 min, a flow rate of 0.3 mL min^−1^, and a mobile phase composed by MeOH and ultrapure H_2_O. The gradient flow was: 0-8 min [H_2_O/MeOH60:40], 8.5 min [H_2_O/MeOH; 56:44], 14.5 min [H_2_O/MeOH; 46:54], 15 min of [MeOH100%], 15.5-17 min, [H_2_O/MeOH; 60:40]. An Acquity UPLC BEH C18 column (Ø = 1.7 μm, 2.1 mm X 50 mm) was used. Data were taken using Masslynx V4.1 software (Waters^®^).

Additionally, using leaf samples of Embrapa 48 and accession PI230970 of three-week-old were analyzed for identification of the Volatile Organic Compounds (VOC's). These plants were maintained in glass container (60 cm high x 15 cm internal diameter), opened at the bottom and with two collection openings, one lateral for air entry and one at the top (for outlet) to VOC acquisition according the standard procedure of Webster *et al.* (2008). These experiments and GC analyses were carried out at Rothamsted Research Experimental Unit, Harpenden – UK.

The samples were analyzed by gas chromatography (GC) (Agilent 6890). Conditions were as follows: The oven temperature was maintained at 30 °C for 1 min, and set at 5 °C/min to 150 °C, and maintained for 0.1 min, and then 10 °C/min to 250 °C. Four microliters of each eluted sample were injected into the GC. Data were analyzed using HP ChemStation software. The quantification of VOCs was standard samples (Skelton *et al.*, 2010). The gas chromatography-mass spectrometry (GC-MS) analysis was performed using a fused silica capillary column (50 m x 0.32 mm thickness, 0.52 μm thick film, DB-1), connected to a temperature-controlled injector. Ionization was by electron impact (70 eV, temperature 250 °C). Helium was the carrier gas. The oven temperature was maintained at 30 °C for 5 min, and then set at 5 °C per min to 250 °C. Identifications were made by comparing the spectra with the spectral mass database (NIST, 2005).

## Results

### *In silico* analysis of genes involved in the biosynthesis of terpene and glyceollin precursors during pathogen infection

We conducted an *in silico* analysis of soybean public transcriptome data available for 13 model genes identified in the MVA pathway (Table S3), aiming to infer the involvement of genes related to terpenes production, derivated by the MVA pathway, in soybean defense mechanisms in response to different biotic stresses, in particular by *P. pachyrhizi*. The transcriptome analysis allowed the identification of nine studies, where at least one of 13 enzymes was differentially expressed, in at least one treatment in response to different pathogens, in at least three studies of soybean responses to infection with *P. pachyrhizi* (Table S3).

Based on transcriptome data, the main enzymes of the MVA pathway had the same expression profile for most interactions. For example, up-regulation of isopentenyl-diphosphate Δ-isomerase and geranyl diphosphate synthase and down-regulation of diphosphomevalonate decarboxylase (Glyma.10g426300) were observed.

It was possible to observe a differential expression of all 13 evaluated genes in at least one of the experiments involving the genotypes harboring *Rpp1*, *Rpp3* and *Rpp4* genes, with only a subset of the enzymes (mevalonate kinase, phosphomevalonate kinase, (2E, 6E)-farnesyl-diphosphate synthetase and geranyl-geranyl diphosphate synthase) displaying down-regulation in the periods evaluated. From all significant expression changes, around 18% correspond to the *P. pachyrhizi* and soybean interaction, independent whether the interaction was compatible or incompatible (p < 0.05, fold-change ≥ 1.0, ratio ≥ 0.5). The levels of expression (fold-change) ranged from −95.14 (Glyma01g42450, hydroxymethylglutaryl-CoA synthase) to 34.66 (Glyma17g05500, (*E,E*) -α-farnesene synthase). The differential expression of the genes encoding the enzymes mevalonate kinase, phosphomevalonate kinase, (2E, 6E) farnesyl-diphosphate synthase and geranyl diphosphate synthase was detected in all three experiments involving soybean backgrounds containing the resistance genes. All significant expression changes are summarized in Table S3.

Some of the genes studied presented a similar expression profile under different pathogen infections, such as the induction of isopentenyl diphosphate isomerase and geranyl diphosphate synthase during infection with *H. glycines* (Ithal *et al.*, 2007)*, P. sojae* (Tyler, 2007), aphids (Studham and Macintosh, 2013) and *P. pachyrhizi* (Choi *et al.*, 2008); and down-regulation of diphosphomevalonate decarboxylase during *P. sojae* infection (Zhou *et al.*, 2009). In addition, the same gene expression profile was observed in soybeans after *P. pachyrhizi* and *P. sojae* infection, and differential expression was shown for all 13 evaluated genes.

### Expression profiles of genes involved in the biosynthesis of terpene and glyceollin during *P. pachyrhizi* infection

**Figure 2:**
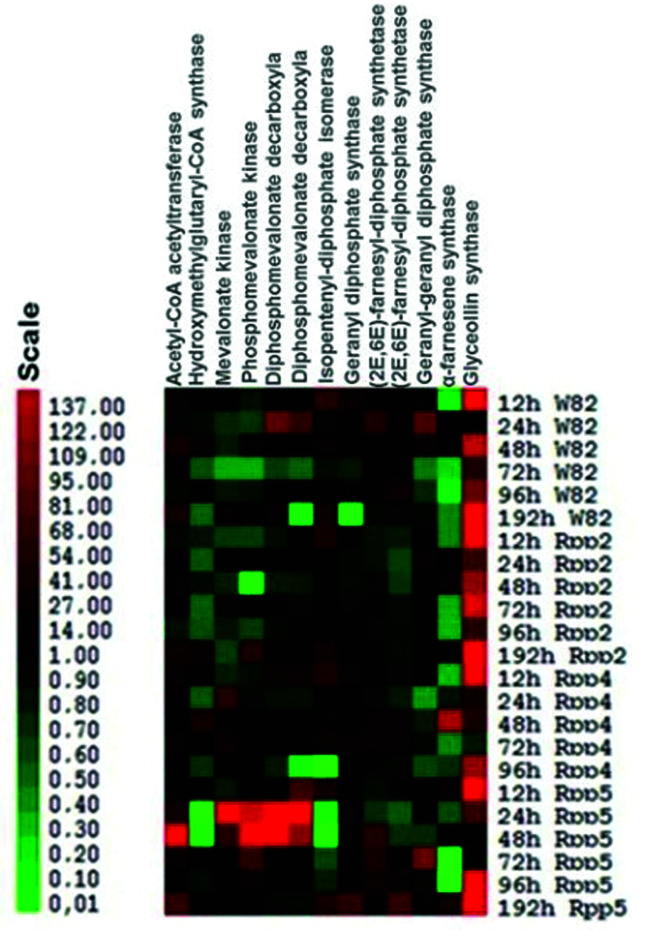
Expression profiles of the genes involved in terpenoid and glyceollin biosynthesis in soybean after infection with *P. pachyrhizi*. Expression values are presented as the log2 of the RQ (relative quantification) values determined by the Software REST (Relative Expression Software Tool) (Pfaffl *et al.*, 2002). These values were converted into a colorimetric scale by the Cluster and Tree View software. Legend: W82 refers to the susceptible cultivar Williams 82, Rpp2 to the resistant PI 230970, Rpp4 to PI459025 and Rpp5 to PI200487. Enzymes: 2.3.1.9, acetyl-CoA acetyltransferase; 2.3.3.10, hydroxymethylglutaryl-CoA synthase; 2.7.1.36, mevalonate kinase; 2.7.4.2-phosphomevalonate kinase; 4.1.1.33, diphosphomevalonate decarboxylase; 5.3.3.2, isopentenyl diphosphate isomerase; 2.5.1.1, geranyl diphosphate synthase; 2.5.1.10, (2E,6E)-farnesyl diphosphate synthase; 2.5.1.29, geranyl-geranyl diphosphate synthase; 4.2.3.46, α-farnesene synthase (GmTPs); 2.5.1.36, glyceollin synthase. The color scale can be observed below the figure corresponds to the interval between fold-change values.

According to the RT-qPCR analysis, in the susceptible genotype Williams 82 (W82), most of the genes that participate in IPP production were repressed or not differentially expressed (Figure 2). Only the enzymes acetyl-CoA acetyltransferase, hydroxymethylglutaryl-CoA synthase and diphosphomevalonate decarboxylase were induced specifically at 48 hai and 192 hai. In addition, isopentenyl-diphosphate Δ-isomerase was induced at 12 and 192 hai. At 72 hai, there was already intense down-regulation of the enzymes acetyl-CoA acetyltransferase, hydroxymethylglutaryl-CoA synthase, mevalonate kinase and phosphomevalonate kinase. All prenylases were induced during at least one of the timepoints evaluated. In this genotype, up-regulation of the enzyme (*E,E*) -α-farnesene synthase (GmTPS) was not observed; instead, a strong downregulation of this gene was observed.

In the *Rpp2* genotype, a slight up-regulation of the enzyme acetyl-CoA acetyltransferase was observed, while the other genes were repressed or not differentially expressed, with the exception of the later up-regulation of the enzyme isopentenyl-diphosphate Δ-isomerase. The prenylases enzymes geranyl diphosphate synthase and geranyl-geranyl diphosphate synthase were induced in 12 and 24 hai, respectively, even after significant down-regulation of genes encoding MVA pathway enzymes and, consequently, the IPP levels. There was considerable down-regulation of (2E, 6E)-farnesyl-diphosphate synthetase at all evaluated timepoints.

In the *Rpp4* genotype, slight up-regulation of hydroxymethylglutaryl-CoA synthase, mevalonate kinase, diphosphomevalonate decarboxylase and isopentenyl-diphosphate Δ-isomerase was observed at specific timepoints. However, we observed up-regulation of prenylases involved in the formation of FPP precursors (geranyl diphosphate synthase and (2E, 6E)-farnesyl-diphosphate synthetase), followed by up-regulation of GmTPS (*E,E*) -α-farnesene synthase - E.C. 4.2.3.46). Significant down-regulation of geranyl-geranyl diphosphate synthase was observed. It was also observed up-regulation of glyceollin synthase involved in production of glyceollin at all timepoints studied.

In the *Rpp5* harboring genotype, differently from the other *Rpp* sources, the pathway was strongly induced, except for the enzyme hydroxymethylglutaryl-CoA synthase. The strongest up-regulation was observed in the enzymes phosphomevalonate kinase and diphosphomevalonate decarboxylase, which reached expression levels between 79.8 and 137 times more induced in inoculated samples compared to the false-inoculated samples at 48 hai. At the same timepoints, strong down-regulation of isopentenyl-diphosphate Δ-isomerase was observed, coinciding with the up-regulation of prenylases enzymes geranyl diphosphate synthase and (2E, 6E)-farnesyl-diphosphate synthetase. At 12 hai, the enzyme geranyl diphosphate synthase was upregulated in all genotypes, except in the *Rpp5* genotype.

The enzyme glyceollin synthase was intensely induced at almost all timepoints and in all genotypes evaluated after inoculation with the fungus. However, GmTPS involved in the production of sesquiterpene (*E,E*) α-farnesene (E.C. 4.2.3.46) was generally repressed after *P. pachyrhizi* infection, with the exception of the expression pattern observed in the *Rpp4* background. All results can be found in Figure 2 and Table S2.

### Glyceollin and (E,E) α-farnesene levels after *P. pachyrhizi* infection

**Figure 3:**
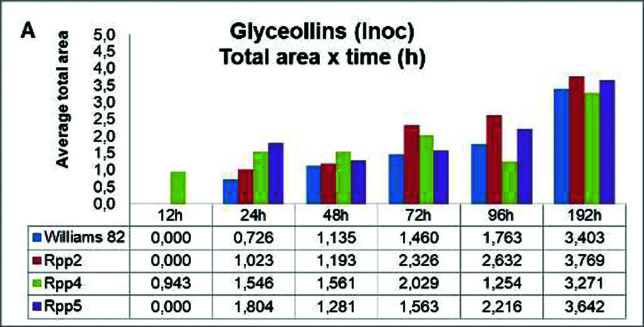
Glyceollin concentrations in soybean leaves of different genotypes [Williams 82, PI 230970 (Rpp2), PI 459025 (Rpp4), PI 200487 (Rpp5)] inoculated with *Phakopsora pachyrhizi*, evaluated at six time periods (12, 24, 38, 72, 96, and 192 hai). Data represent the mean of three replicates of representative inoculated samples.

Inoculation of soybean with the fungus *P. pachyrhizi* showed a glyceollin peak in all genotypes tested, starting at 24 hai, but at this time, the susceptible genotype presented the lowest glyceollin level. In general, the accumulation of phytoalexin was increasing across the infection cycle. At 192 hai, all genotypes presented nearly near the same levels of glyceollins, as revealed by the peak area (Figure 3).

**Figure 4:**
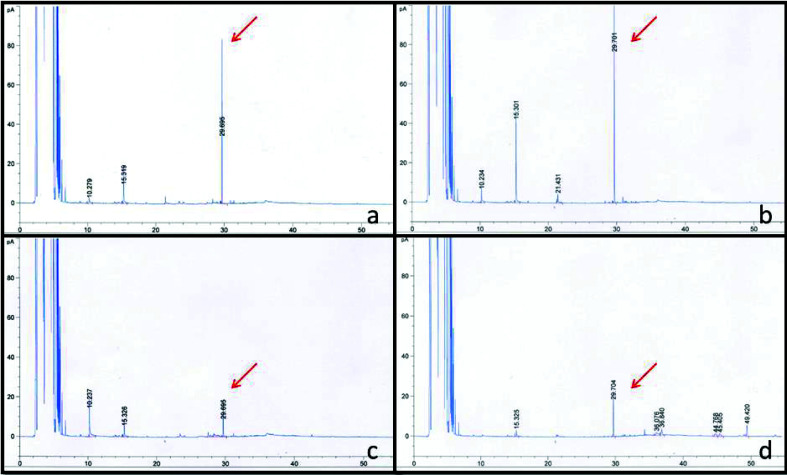
Chromatographic profiles of volatile solutions obtained from soybean plants, Embrapa 48 (a, c) and PI 230970 (b, d) genotypes. The arrow indicates the volatile compound that had its emission reduced after inoculation with the fungus. (a, b) False-inoculated soybean plants. (c, d) Soybean plants that were *Phakoposra pachyrhizi* inoculated.

Volatiles collection in *P. pachyrhizi* inoculated soybean was started 18 hai and passed over 48 hours, totalizing 66 hai of collecting process. It was observed that after soybean infection with *P. pachyrhizi* fungus, one of the VOCs emitted by the plant had its production reduced, compared to false inoculated plants, in both resistant and susceptible genotypes (Figure 4).

Comparing the spectra obtained with the spectral mass database, the reduced compound was identified (over 70% probability) as the metabolite (*E,E*) α-farnesene, that was later confirmed by a co-injection of the VOC solution and the external standard (E, E) α-farnesene. The peak obtained on the chromatogram was unique at the retention time corresponding to (*E,E*) α-farnesene.

The plants were also evaluated daily for rust symptoms. Disease symptoms appeared around seventh day after inoculation. After comparing the chromatographic profiles, no other compounds were associated with the presence of *P. pachyrhizi* in the infected leaves. Phenotypic analysis showed that high level of disease symptoms was related to higher (*E,E*) α-farnesene reduction among infected plants at the initial times of infection evaluated (Table 2).

**Table 2:** Percentage of infected leaf area in *P. pachyrhizi* inoculated soybean plants and the production of (E, E) alpha-farnesene over 48 hours.

Embrapa 48			PI 230970		
*Rep*	*% AFI*	ng/μL *(E ,E)* α*-farnesene*	*Rep*	*%* AFI	pA (E ,E) α-farnesene
1	7.05	5.13	1	4.24	3.25
2	5.55	1.95	2	1.20	7.03
3	0.34	12.50	3	0.18	24.63
4	8.61	3.64	4	1.70	8.71

Tests performed with detached soybean leaves inoculated with *P. pachyrhizi* did not allow us to infer any direct effect of volatile (*E,E*) α-farnesene in the infectious process. Leaves developed disease symptoms characterized as TAN lesions (Embrapa 48) and RB-type lesions (PI 230970), both in which solutions were added (*E,E*) α-farnesene as in used as control. However, further testing needs to be performed, especially regarding an efficient dilution of the compound (results not shown).

## Discussion

### Genes involved in terpene and glyceollin biosynthesis are induced by different soybean pathogens

The MVA pathway consists of six enzymatic reactions initiated by the condensation of three acetyl-CoA units, forming 3-hydroxy-3-methylglutaryl-CoA, which is reduced to mevalonic acid, followed by two additional steps of phosphorylation and decarboxylation, culminating with the formation of IPP, the main precursor for the formation of terpenoids in the cytoplasm. Six genes encoding enzymes of the MVA pathway, culminate with the production of IPP precursor. The transcript levels of the enzyme isopentenyl diphosphate isomerase (IDI), which is related to the conversion of IPP to DMAPP, were also analyzed here, once DMAPP is an important precursor of glyceollin, by the action of the glyceollin synthase (Figure 1).

From IPP, terpene production in plants is initially dependent of the prenylases (geranyl diphosphate synthase, geranyl-geranyl diphosphate synthase and (2*E*,6*E*)-farnesyl diphosphate synthase), responsible to convert IPP into GPP (geranyl diphosphate), FPP (farnesyl diphosphate) and GGPP (genranyl-geranyl diphosphate); and later by different terpene synthases, that use these precursors as substrates for monoterpenes (10 carbons), sesquiterpenes (15 carbons) and diterpenes (20 carbons) production. The enzyme GmTPS (*E,E*)-α-farnesene synthase acts in the final step of (*E, E*) -α-farnesene (a sesquiterpene) production, converting FPP or GGPP into (*E,E*)-α-farnesene (Figure 1).

The activation of genes of the MVA pathway, prenylases, and glyceollin and terpene synthases in soybean (GmTPS) was observed in different soybean-pathogen interactions on the transcriptome studies, corroborating that these pathways are broadly activated by biotic stress in soybean, as part of the basal resistance response. Production of these compounds may not require specific recognition by the pathogen.

Based on the transcriptome data available, the prenylases involved in the synthesis of mono-, di- and sesquiterpenes and glyceollin were activated by *P. pachyrhizi* infection, but was not specific for this pathogen. The data also did not reveal a pattern of activation or down-regulation of a specific pathway in a specific pathosystem. We observed only five genes differentially expressed in response to a specific pathogen, including the enzymes acetyl-CoA acetyltransferase, diphosphomevalonate decarboxylase, geranyl diphosphate synthase, (2*E*,6*E*)-farnesyl diphosphate synthase and geranyl-geranyl diphosphate synthase in response to aphids. Similarly, acetyl-CoA synthase was specifically upregulated in experiments where soybeans were infected with *P. sojae* (Tyler, 2007; Studham and Macintosh, 2013) and aphids (Zhou *et al.*, 2009).

### Terpene biosynthesis is part of *P. pachyrhizi* defense response in soybean and is dependent of the *Rpp* background in soybean genotypes

In general, we observed a slight activation of the MVA pathway in the susceptible (W82) and in the *Rpp4* genotypes in response to *P. pachyrhizi* until IPP production*.* In the *Rpp2* background, all genes evaluated in MVA pathway were strongly repressed, while in *Rpp5* genotype, a completely opposite pattern was observed, with a strong induction of the pathway. Thus, considering the expression data obtained in this study, terpene production may also contributes to resistance response to *P. pachyrhizi*, but being temporal regulated depending on the *Rpp* background,

Most genes in the W82 genotype leading to the production of IPP were either repressed, and it was possible to observe a discrete up-regulation during haustorial development (48 hai) of three genes (acetyl-CoA acetyltransferase, hydroxymethylglutaryl-CoA synthase and diphosphomevalonate decarboxylase). At 192 hai, which corresponds to the uredia formation, four genes were induced (acetyl-CoA acetyltransferase, hydroxymethylglutaryl-CoA synthase, diphosphomevalonate decarboxylase and isopentenyl diphosphate isomerase). However, strong down-regulation was observed at 72 hai, where four genes (acetyl-CoA acetyltransferase, hydroxymethylglutaryl-CoA synthase, mevalonate kinase and phosphomevalonate kinase) were significantly repressed, and the other genes were not differentially expressed, which is characteristic of a biphasic response, with two up-regulation peaks interspersed by a strong down-regulation of gene expression (Figure 2, Table S2).

A type of biphasic expression pattern in response to rust had already been reported in the Embrapa 48 genotype, and in this genotype, two peaks of up-regulation were observed at 12 and 96 hai. In the *Rpp2* genotype, expression peaks occurred at 12 and 72 hai. The second phase of up-regulation of resistance response was faster in resistant genotype, coinciding with the stages of haustorium formation and consequent secretion of proteins involved in virulence of fungi and was indicated to be crucial to the resistance phenotype (van de Mortel *et al.*, 2007). It should be noted, however, that the biphasic response presented in that study was mainly related to activation of type MYB and WRKY transcription factors, potentially regulating the production of phenylpropanoids and lignin precursors. These pathways were also activated in the *Rpp3* and *Rpp4* genotypes (Meyer *et al.*, 2009; Schneider *et al.*, 2011), and a biphasic response to fungus was also described. Subsequently, transient silencing in soybean (Pandey *et al.*, 2011) functionally validated the involvement of such pathways in the resistance response to rust in the *Rpp2* genotype. Thus, the production of phenylpropanoid secondary metabolites may be one of the main responses to *P. pachyrhizi* infection in soybean, as it is observed in different *Rpp* resistance genes sources. The terpene production may also contribute to resistance response to *P. pachyrhizi*, however a similar pattern of activation was not observed among the genotypes harboring different *Rpp* genes.

Interestingly, even though we could not observe a similar pattern in the MVA pathway among the different *Rpps* sources, at least the prenylase geranyl diphosphate synthase was up-regulated in all genotypes assessed at initial timepoint after infection (12 hai), returning to basal levels until 192 hai, when it was significantly induced again, coinciding with the penetration and sporulation stages of the *P. pachyrhizi* infection cycle, demonstrating that at least the formation of monoterpens precursors is common among the genotypes and its production might be associated with the initial and late steps of infection. The *Rpp*5 genotype was the only one where this biphasic pattern was not observed (Figure 2, Table S2).

The Rpp4 genotype likewise W82, displayed a slight up-regulation of four MVA pathway genes evaluated (hydroxymethylglutaryl CoA synthase, mevalonate kinase, diphosphomevalonate decarboxylase and isopentenyl diphosphate isomerase) at specific timepoints. A biphasic response was not observed (Figure 2, Table S2). It should be noted that, as in W82 genotype, the enzyme isopentenyl diphosphate isomerase, responsible for reversible reaction of IPP to DMAPP, was induced in Rpp4 genotype at the initial timepoints. This shows that even with slight production of IPP by the MVA pathway in these genotypes, the IPP may still be converted into DMAPP and, consequently, may be involved in other terpenoids production, such as mono- and diterpenes, as demonstrated by expression profile of different prenylases observed in this study in this genotype.

In susceptible W82 genotype, all genes encoding prenylases were induced at least at one evaluated time point, indicating that mono-, di- and sesquiterpenes may be relevant to development of basal defense mechanisms of hosts ASR infected. However, in this genotype, there was no up-regulation of enzyme (*E,E*) -α-farnesene synthase (GmTPS); instead, a strong down-regulation of this gene was observed (Figure 2, Table S2), suggesting that there is no (*E,E*) -α-farnesene production in compatible reactions, or in lower levels, corroborating with results of VOCs detection in susceptible genotype Embrapa 48 after rust infection (Figure 4, Table 2).

In *Rpp*4 genotype, the up-regulation of prenylases associated with FPP precursor formation (geranyl diphosphate synthase and (2E, 6E)-farnesyl diphosphate synthase) establishment was observed, followed by up-regulation of GmTPS (*E, E*) -α-farnesene synthase), indicating the possible production of (E, E), α-farnesene, at least during haustorial development and secretion of effector proteins (48 hai). Additionally, significant down-regulation of geranyl-geranyl diphosphate synthase was observed, indicating “capture” of the FPP precursor, favoring production of sesquiterpenes over diterpenes (Figure 2, Table S2). Choi *et al.* (2008) also observed the up-regulation of (*E,E*) -α-farnesene synthase in a genotype containing the *Rpp*1 (PI200492) resistant-gene, after infection with two different isolates (HW94-1 and TW72-1) at the same timepoint of 48 hai (Table S2).

Different from the Rpp4 and W82 genotypes, *Rpp2* genotype displayed strong down-regulation of all MVA pathway genes evaluated in response to *P. pachyrhizi*. A slight up-regulation was only observed in first enzyme of the pathway (acetyl-CoA acetyltransferase) at 24 hai and at final timepoint evaluated (192 hai). Tremblay *et al.* (2011) also verified the expression profile of this enzyme in W82 and Rpp2 genotypes, with a strong up-regulation at 48 hai, a crucial period for fungal development, when the pathogen has established many haustoria and is secreting effector proteins to interrupt the defense mechanisms plant. At all other time-points evaluated in this study, all genes were significantly down-regulated or were not differentially expressed (Figure 2, Table S2). Repression of MVA pathway in *Rpp2* genotype may compromise the production of IPP, the main precursor for terpenoid production in cytoplasm. This result was demonstrated by evaluation of the VOC sesquiterpene (*E,E*) α-farnesene production. This sesquiterpene was repressed after *P. pachyrhizi* infection in both, susceptible Embrapa 48 genotype and a resistant genotype containing *Rpp2* gene (Figure 4, Table 2).

Alternatively, IPP can also be produced by plastidial MEP pathway (Akashi *et al.*, 2009). Thus, the cytoplasmic MVA pathway appears to be out of the synthesis of this important terpenoid precursor in the *Rpp2* background, indicating that down-regulation of cytoplasmic pathway may be the cause of reduction in VOC levels in the PI230970 genotype (Figure 4 and Table 2). Although a strong down-regulation of MVA pathway was observed, the up-regulation of isopentenyl diphosphate isomerase, which connects the two pathways involved in IPP production, may be an indicative of IPP supply by plastidial MEP pathway, ensuring the presence of prenyl precursors for terpenoid formation in cytoplasm.

The prenylases enzymes geranyl diphosphate synthase and geranyl-geranyl diphosphate synthase, involved in the formation of mono and diterpenes, were induced at 12 and 24 hai, respectively, in initial timepoints of fungal development, even after significant down-regulation of MVA pathway in this genotype, suggesting again that IPP precursors are potentially derived from the MEP pathway. The considerable down-regulation of (2E, 6E)-farnesyl diphosphate synthase at all timepoints evaluated in this genotype suggests that mono- and diterpenes are being preferentially generated in relation to the development of (*E,E*)-α-farnesene (Figure 2).

Distinct from other genotypes evaluated in this study, the genotype containing the *Rpp5* resistance gene displayed strong up-regulation of MVA pathway throughout the infection cycle of *P. pachyrhizi*. With exception of the gene encoding hydroxymethylglutaryl-CoA synthase, all genes involved in IPP production were strongly upregulated, mainly at the timepoints corresponding to haustorial development (24 and 48 hai). Interestingly, at 48 hai, a strong down-regulation of isopentenyl-diphosphate isomerase was also observed, suggesting a possible accumulation of IPP. At this time, the pathogen was able to establish plant tissue colonization with haustorium formation and, consequently, direct contact with effector secretion in plant cytoplasm. The strong up-regulation of MVA pathway suggested immediate detection of fungus by this genotype and the potential importance of terpenoid synthesis in resistance response mediated by *Rpp5*. The repression of the prenylase isopentenyl diphosphate isomerase at intermediate timepoints, coinciding with up-regulation of the enzymes geranyl diphosphate synthase and (2E, 6E)-farnesyl diphosphate synthase, indicated that mono- and diterpene synthesis is preferentially occurring in this genotype.

Based on *in silico* and *in vivo* analyses, it is possible to conclude that there is a group of terpenes produced by plant depending on the type of biotic stress. In the case of soybean response to *P. pachyrhizi*, we observed a differential induction of genes involved in terpenes production dependent on *Rpp* genotype. However, we could not observe a significant difference between susceptible and resistant genotypes studied, indicating that (*E,E*)-α-farnesene and other terpenes are involved in plants basal defenses, in general.

### GmTPS involved in the production of the sesquiterpene (*E,E*)-α-farnesene is generally repressed after *P. pachyrhizi* infection

The sesquiterpene (*E,E*)-α-farnesene production in plants is derived from FPP or GGPP precursors. The enzyme GmTPS (*E,E*)-α-farnesene synthase acts in the final step of volatile α-farnesene, converting the precursors into (*E,E*)-α-farnesene.

A strong down-regulation of prenylase (2*E*,6*E*)-farnesyl diphosphate synthase was verified, followed by GmTPS down-regulation in *Rpp2* genotype, confirming that in this genotype, the precursors generated were potentially used in diterpene synthesis, and subsequent (*E,E*) -α-farnesene reduction observed by VOC levels.

In the susceptible genotype (W82), although FPP had been synthesized, significant GmTPS down-regulation was observed, suggesting that in this genotype, FPP was used as a substrate for mono- and diterpene production.

In the *Rpp2* (PI230970) accession and in the susceptible genotype Embrapa 48, we could demonstrate the reduction of levels of the VOC (*E,E*) -α-farnesene across the infection cycle, corroborating with the expression profile of the genes in MVA pathway, the prenylases and GmTPS observed. Interestingly, the (*E,E*) -α-farnesene production was negatively associated with the *P. pachyrhizi* symptoms at least in these two genotypes. The reduction of the VOCs levels could be detected before the symptoms appearance.

GmTPS up-regulation was only observed in Rpp4 background and was coordinated with prenylases activation involved in FPP formation, indicating that the VOC (*E,E*) α-farnesene may be produced at least at specific time of 48 hai in this genotype. Additionally, the down-regulation of the enzyme geranyl-geranyl diphosphate synthase may be a strategy to save the prenyl precursor FPP, repressing diterpene production and contributing to sesquiterpene production.

Finally, the strong up-regulation of the MVA pathway in Rpp5 followed by up-regulation of the enzymes geranyl diphosphate synthase and (2E, 6E)-farnesyl diphosphate synthase and no activation of GmTPS, indicated that mono- and diterpene synthesis might be preferentially occurring in this genotype.

Further studies to determine the levels of the VOC (*E,E*) α-farnesene in these genotypes (Rpp4 and Rpp5) are necessary to confirm the expression profile results.

### Glyceollin levels are strongly induced by *P. pachyrhizi* infection

The enzyme glyceollin synthase, also known as (-)-glycinol 4-dimethylallyltransferase (G4DT), was dramatically induced after fungal inoculation at almost all time points and in all genotypes evaluated (Figure 2, Table S2), independent of which prenylases associated with different classes of terpenes were preferentially induced. Induction of glyceollin production has been reported in soybean in response to infection by *P. pachyrhizi* (Choi *et al.*, 2008; Pandey *et al.*, 2011; Schneider *et al.*, 2011).

The induction of the glyceollin synthase expression was consistent with the gliceollin levels. The inoculation of soybean leaves with *P. pachyrhizi* fungus resulted in an increase in the peak area for glyceollin in all genotypes tested in comparison to the control (mock-inoculated). The glyceollin level was detected from 24 hai, culminating with the peak area at 192 hai (Figure 3). There was no significant difference between genotypes containing resistance genes and susceptible genotypes; the peak area of phytoalexin was similar in the W82, *Rpp2* and *Rpp5* genotypes. These results are in agreement with the observations of Lygin *et al.* (2009), also studying the same pathosystem interaction (soybean and Asian soybean rust), observed accumulation three weeks after inoculation, especially in resistant genotypes *Rpp*1, 2 and 3.

The higher levels of glyceollin detected only in later timepoint suggested a possible posttranscriptional control mechanism, such as stability, transport or maturation. However, little is known regarding posttranscriptional factors, and the relationship between glyceollin synthase gene expression and effective glyceollin production remains elusive.

There are also reports of soybean phytoalexin production in other soybean interactions and microorganisms, such as with the fungi *P. sojae, P. citricola* (Gijzen *et al.*, 2009; Zhou *et al.*, 2009), and *B. japonicum* (Gijzen *et al.*, 2009; Zhou *et al.*, 2009; Libault *et al.*, 2010; Silva, 2013) (Table S3). These results confirm the importance the glyceollins in basal plant defense mechanisms (Akashi *et al.*, 2009).[Bibr B37]

Therefore, considering the slight up-regulation of the MVA pathway after infection in genotypes evaluated, with the exception of the *Rpp2* genotype, it is possible to infer an unlikely involvement of cytoplasmic pathway in generation of precursors for glyceollin biosynthesis. Only *Rpp5* genotype showed a dramatic up-regulation of MVA pathway, thus being able to generate precursors for glyceollin production by different prenylases action, including G4DT. However, it should be noted that, in this genotype, a strong down-regulation of isopentenyl diphosphate isomerase was also observed, indicating no conversion of IPP to DMAPP. Since high levels of G4DT expression were also observed in *Rpp5* genotype, it is possible infer that the supply of DMAPP to glyceollin production came from MEP pathway, corroborating the results of Akashi *et al.* (2009).

The down-regulation of some genes encoding MVA pathway enzymes observed in the W82, *Rpp2* and *Rpp4* genotypes is consistent with the expression profile observed in the *in silico* analysis, as most of these genes were down-regulated in different plant-stress interactions (Table S3). It is possible that the gene down-regulation observed in these genotypes is a plant mechanism to concentrate energy expenditure to ensure glyceollin production, an important phytoalexin in soybean with great influence in host defense reactions. Similar observations in this study, the *in silico* analysis found that the enzyme isopentenyl diphosphate isomerase was induced at all timepoints and stresses studied, indicating the importance of this enzyme in plant defense mechanisms and connecting IPP production between the cytoplasm and plastids, and providing the cytoplasm with alternative source of IPP.

Based on the observed transcript expression profile and glyceollin levels peak, it is possible to suggest that this phytoalexin has an important role in basal resistance response to *P. pachyrhizi* infection in soybean. Even though, when compared to mRNA levels, protein elevation displays a slower response after detection.

## Supplementary material

The following online material is available for this article:

Table S1 Primers designed and used in this study.

Table S2 Expression profile of soybean genes after *P. pachyrhizi* infection.

Table S3 Transcriptional characterization of genes in response to different soybean diseases.
