# Supplementary material for: Transcriptional profile of genes involved in the production of terpenes and glyceollins in response to biotic stresses in soybean
Source: Genet Mol Biol. 2020 Nov 6;43(4):e20190388. doi: 10.1590/1678-4685-GMB-2019-0388 (PMC7644969; doi:10.1590/1678-4685-GMB-2019-0388)
Supplement: Supplementary file 1 [file 1415-4757-GMB-43-4-e20190388-suppl1.pdf]

Genetics and Molecular Biology
DOI: https://doi.org/10.1590/1678-4685-GMB-2019-0388

Supplementary Material to "Transcriptional profile of genes involved in the production of terpenes and glyceollins in response to biotic stresses in soybean"

Table S1 - Primers designed and used in this study.

Gene ID	Number EC	Glyma	Primer F	Tm	Primer R	Tm	Amplicon
Geranyl 2P synthase	**2.5.1.29**	Glyma.11g063900	GTACGGAAGGGCTTGTGG	59,1	ATGAATTCCAGCGTCTCCAG	60,2	85 bp
Diphosphomevalonate decarbosylase	**4.1.1.33**	Glyma.10g279800	TGGGTATTCATGGTGACTGC	59.4	CCGGTAAGATCAAGGTTTCG	59.6	88 bp
Diphosphomevalonate decarbosylase		Glyma.20g109900	TTCAGATGATGGCGAGTGAG	59.9	GCAATGTTGGTTGGTGTCTG	60.0	70 bp
Phosphomevalonate kinase	**2.7.4.2**	Glyma.06g127200	GCATCTGCAACAAGCACAAC	60.5	ACCGGTCATAAGCACTTTCC	59.1	72 bp
Mevalonate Kinase	**2.7.1.36**	Glyma.03g239000	ACGCATTCCACATGGATCTC	60.9	CCGTTGCTGCATTCAAATC	60.2	70pb
Mevalonate Kinase		Glyma19g424400	CCATGAGGGCAAGAATTGG	61.4	CCATGAACCACAGCATGTTC	60.0	142pb
Hydroxymethylglutaryl-CoA synthase	**2.3.3.10**	Glyma.01g215500	TTTGACTCACGCTGAGAACG	60.2	GCGAAGTTGACTGCTGATTG	59.6	88 bp
Hydroxymethylglutaryl-CoA synthase		Glyma16g324800	ACACACACACCACAACTCTTCC	60.0	GGGCCTGTTTGGATTTAGAG	58.6	123pb
Hydroxymethylglutaryl-CoA synthase		Glyma17g141500	TGGCAAGACCCGGTAAATAG	60.0	GAGGTCGTTAGCTTCGCAAC	60.0	91 bp
Acetyl-CoA acetyltransferase	**2.3.1.9**	Glyma14g007600					
Acetyl-CoA acetyltransferase		Glyma.17g005300	TGAGGCAGAAGAGAGGGAAG	59.7	AAAGAACGTCCCACAGCAAC	60.2	106pb

Genetics and Molecular Biology
DOI: https://doi.org/10.1590/1678-4685-GMB-2019-0388

Taqman			
**Gene ID**	**Local da sonda**	**Marcation**	**Gene sequence**
MV_Gm.01g 215500	1695-1720	FAM	CACCATTTCATTATTGCTTGAACAAGATTGATACACATTCTATTCAAACAGACGCCCATTAAAATTTCCGCCAAAAAAGGA<br>GGGTTAGTTAGGAAGACACACACACACCACAACTCTTCCTAAACCTAACACACACACACTTACACTTTGCATTCCAAATT<br>CCACAACTTTCACTTTCTACAACACTATTTACCCCATTTTCTCTAAATCCAAACAGGCCCCAAAAGAAAGAGAGGGAAAAT<br>TCTCTTCCATGGCCTCTTCACGCCCTGCCAATGTGGGAATCCTTGCCATGGACATCTACTTCCCTCCCACCTGCGTCAC<br>CCAGGATGCTTTGGAGGGTCATGATGGGGTGAGCAAAGGGAAATATACTATTGGGCTTGGACAGGATTGCATGGCCTT<br>CTGCTCTGAGGTTGAAGATGTTATCTCAATGAGCTTGACGGTAGTTACTTCACTTCTTGAAAAATTTAATGTTGATCCAAA<br>GCAAATTGGACATTTGGCGGTTGGGAGTGAAACTGTTATTGACAAAAGCAAATCAATTAAGACCTTCCTGATGCAAGTTT<br>TTGAGGCAAGTGGTAATACTGACATTGAAGGTGTTGATTCAACTAATGCATGCTATGGAGGAACGGCTGCTTTGTTCAAC<br>TGTGTGAATTGGGTGGAGAGTAGCTCATGGGATGGACGTTATGGACTTGTTGTTTGTACAGACACTGCGGTATATGCTG<br>AAGGACCTGCTCGTCCCACTGGAGGAGCTGCTGCAATTGCCATGCTTGTAGGGCCAGATGCTCCTATTGCTTTTGAAAG<br>CAAACTCAGAGGCAGTCACATGTCTCATGCATATGATTTTTACAAGCCAAACCTTGCTAGCGAATATCCAATTGTTGATG<br>GAAAACTCTCACAGACCTGTTATCTCATGGCACTTGATTCCTGTTACCGGCTTTACTGTGAGAAATTTGAAAAATTGGAG<br>GGGAGGCCTTTTTCAATGTCAGATTCTGATTATTTTGTGTTTCATTCTCCATATAACAAGCTTGTGCAGAAAAGTTTTGGC<br>CGACTATACTTCAATGACTTCTTGAGAAATGCCAGTTTTGTTGATGAAGTTGCCAGGGAAACCCTTGCACCATATGCATC<br>CTTATCTGGTGATGAGAGTTATCAAAGTCGTGATCTTGAAAAGGCAAACCAGCAAGCTGCAAAACATCTATATGATGCAA<br>AGGTGCAGCCCAGCACACTAATCCCAAAGCAAGTTGGTAACATGTACACTGCATCTCTTTATGCAGCATTTGCATCTCTT<br>CTTCACAATAAGAACAGTTCGTTGGTAGGTAAACGGGTAGTTATGTTTTCATATGGAAGTGGTTTAACAGCTACAATGTTC<br>TCCTTCCAGCTTCAAGAGGGTCAACATCCGTTTAACTTGTCAAACATTGTAACAGTGATGAATGTTTCGGACAAGTTGAA<br>GCAGAGAGTTGAGATTCCTCCTGAAAAGTTCGTTGAAACATTGAAGATCATGGAACACCGTTATGGGGGTAAGGACTTT<br>GTGACAAGCAAGGACTGTAGCTACTTAACTCCAGGCACCTTCTATCTCACCAATGTTGATTCCATGTACAGGAGATTTTA<br>TGCCAAGAAGGACTAGGTTATTGGTTAGTTGATGATCGCTGATATATTCAAATATGGAGTTAAAATGTATTTTCTTATGGT<br>TCACCTGTATTTGATCCCATAAGGTTGTGTATCAAAATGTTTATAAGATGTTTCATTTTTTTGTGGTGATCAGGTTGTAACC<br>AAAATAAGAATAAGTTTATTTGCGAATTGGCAATTGTTTTTAAATGCACTAATAATCCAAATATATTAGTAGTTTCTTCTTGA<br>GTGCAAAAAAAG

DOI: https://doi.org/10.1590/1678-4685-GMB-2019-0388

Taqman			
**Gene ID**	**Local da sonda**	**Marcation**	**Gene sequence**
MV_Gm.03g 239000	1363-1384	FAM	GGCCGAATGCTGAAAAACGTACAAAAGGTTAGTTGTTAATTAAATGGCGCACGCATTCCACATGGATCTCCTCCACGCT<br>GGACTTTTGGACACAAATACAAGATTTGAATGCAGCAACGGGGAAAGCCTATGAATTAAGATTCAGAGATCATAAGAGTA<br>ATAGAAATTAGAATGGAGGTTAAATCCAGAGCTCCCGGGAAAATTATCCTAACCGGCGAACATGCTGTGGTTCATGGAT<br>CCACCGCTGTTGCTTCTTCTATTGACTTGTATACCTACGTTTCTCTCCACTTCTCCACTCCTTCCGACAACGAGGATTCGT<br>TGAAACTGAAGCTGCAGGAGACGGCGTTGGAGTTCTCGTGGCCAATCACGAGAATAAGAGCAGCGTTTCCTGAATCCA<br>CGGCTCAGCTATCTTCCACGCCGAACTCATGCTCTGTGGAGAATGCCAAGGCAATTGCCGCACTCGTTGAAGAGCTTAA<br>CATTCCAGAGGCCAAACTCGGACTCGCCTCTGGAGTTTCCGCCTTTCTCTGGTTATACTCTTCCATTCAAGGATTTAAGC<br>CTGCTACTGTTGTTGTCACTTCTGAACTTCCTCTGGGCTCAGGATTGGGTTCATCCGCCTCGTTTTGTGTTGCGCTGGCG<br>GCCGCCTTGTTGGCTTATACTGATTCTGTCTCTCTGGATTTGAAACATCAAGGATGGCTCTCCTTTGGGGAGAAGGATCT<br>TGAGTTGGTAAATAAATGGGCTTTTGAAGGGGAGAAGATCATTCATGGAAAGCCCTCTGGAATTGACAACACAGTAAGC<br>GCATATGGTAACATTATCAGCTTCAAGTCGGGTAACTTGACACATATGAAGTCAAGTGTGCCGCTTAAAATGCTCATTAC<br>TAACACCAAAGTAGGGAGGAACACAAAAGCATTGGTGGCTGGTGTTGGAGAGAGGATGCTAAGGCATCCAGATATAATG<br>GCTTTTGTGTTTAGTGCTGTTGATTCTATTAGCAATGAATTGACTTCCATTCTCAAGTCACCTACACCAGATGAGCTCTCG<br>GTAACTGAGAAAGAAGAGAAGATAGAAGAACTAATGGAAATGAATCAAGGTATGCTCCAGTCTATGGGGGTCAGTCATG<br>CCACAATAGAAACTGTTCTTCGAACAACATTGAAGTATAAGTTAGCCTCCAAATTGACAGGAGCTGGTGGTGGGGGCTG<br>TGTTCTGACACTGCTTCCAACATTGCTATCAGGCACCGTTGTTGACAAAGTAGTTGCTGAACTGGAGTCATGCGGGTTCC<br>AATGTTTCATTGCTGGAATTGGCGGGGGAGGTGTTGAAATTAGCTTTGGGGTGTCATCTTGATTTTCTGTTATTATTTTTC<br>CAAGGACTGACATCAATGATCACAGCATCGGATCCTCAAATAATCAGCATAAAAATATATGCTACCCTTTTTACAGCTTGT<br>GCATCAATCTGTTACTTTTTCTTTTCTACTTTTGAATCAATTTAAGCATTTTCTTATTCAATCCTGAACGGAAAATGGAGAAT<br>ATAAACTCATATTTGGTTAATAAGTACCATTCGAGTTTGATCCTTGACGAAAATAATTATTAGTCAAATTTTATTTATCTTCT<br>GGT

Genetics and Molecular Biology
DOI: https://doi.org/10.1590/1678-4685-GMB-2019-0388

Taqman			
**Gene ID**	**Local da sonda**	**Marcation**	**Gene sequence**
MV_Gm.17g 005300	1623-1654	FAM	GTTTTGGCTTGCCCGGATTCATTAAACGGTGCGTGTAACTCGTGGCTCGTCCCACCTGACCTTATATAACCACTCAGATC AGTCGAACATTTCTTCACTTTCGTTTTCGACGATCCCCTTCTCCTTCTCTCTCTCTTTTTCGCCTCATTCCATTTTCATCTA TCAATGGCTCCAGTAGCAGCAGCATCTTCAGATTCAATCAAGCCAAGAGATGTTTGCATCGTTGGTGTTGCACGTACACC AATGGGTGGATTTCTTGGTACTCTGTCATCTCTATCTGCCACCAAGCTAGGCTCTATAGCTATTGAAGCTGCTCTTAAAA GGGCCAATGTTGATCCATCCCTTGTGGAAGAAGTATTTTTTGGGAATGTTCTTAGTGCTAATTTGGGGCAAGCTCCTGCA AGACAAGCTGCTCTTGGAGCAGGAATATCCAATTCAGTTATCTGCACTACCGTTAACAAAGTTTGTGCATCAGGAATGAA AGCTGCAATGCTTGCTGCACAGAGTATTCAATTAGGCACAAATGATGTTGTTGTGGCTGGTGGTATGGAAAGCATGTCTA ATGTACCCAAGTACCTGGCTGAAGCAAGGAAAGGATCACGCCTTGGACATGATTCACTTGTTGATGGGATGTTGAAAGA TGGTTTGTGGGATGTCTATAAGGATGTTGGCATGGGAGTGTGTGCTGAGCTATGTGCAGATAACCATGCATTAACAAGA GACGACCAGGATAACTATGCAATTCAGAGTTTTGAACGTGGAATTGCTGCCCAAGAAAGTGGTGCCTTTTCATGGGAAAT TGCTCCAGTTGAAGTCTCTGGTGGAAGAGGAAGACCATCAACAGTTGTTGATAAGGATGAAGGCCTAGGAAAGTTTGAT GCTGCCAAGTTACGCAAACTTCGGCCAAGTTTCAAGGAGACTGGAGGTTCTGTTACAGCTGGCAATGCTTCCAGCATAA GTGATGGTGCTGCTGCACTAGTTTTGGTGAGTGGAGAGAAGGCACTGAAGCTTGGGCTTCAAGTTATTGCAAAAATCAC TGGATATGCTGATGCTGCTCAGGAACCAGAGTTATTTACAACGGCTCCATCCCTTGCCATTCCCAAAGCTATTGCCAAGG CGGGGTTGGAGACTTCACAAATTGATTTTTATGAAATTAATGAAGCCTTTGCGGTTGTGGCTCTCGCAAATCAGAAACTT CTTGGACTTAACTCGGAAAAAGTAAACGTACATGGTGGAGCTGTTGCACTGGGTCATCCTCTTGGTTGCAGTGGTGCTC GCATTCTGGTGACACTTTTGGGGGTACTGAAGCAGAAGAATGGGAAGTACGGAGTTGGTGGCATTTGCAATGGAGGAG GTGGTGCATCTGCCCTTGTTGTTGAGCTTCAGTAAGACCTATTTCATGTTCAGTAGATGTCCTTTTAAGAGCCAGCGTTT GATGCAAAGCTCAAAAGATCTTGCCTCCCTCTTTAGCAAGCGAACATGCAAGTTTCAAATTTATTACAAGGACAAGACCG AAAGAGAGCGAAATTTAGCTACATAGTTGTTAGGTTTAAGTCGCATCTTGTGAAAGTGAAATTTTCTTGGCATCAAAGTAG AACATGAAACAATATTATCCAAATTTTAGTTTTCTCTGAAATGTTATTGGCGTACTTTGGTTTGGAATGAATCATTATAAAA CCTCTTATTCCAAGCACAAGATTACAGATTCCCGTGGTTTGCAAAATGTGTTCCAGAACTGAAATTGCCAAAAAGAGTGG AAATAAGGAAGTTGTAGTTGGTCTTGCGTTTGCTAATTTAGATCTAACATTTTATTCTGAATATTCTGGCACGCTAAGCAA GTCAAAGTGTTTTCTCCTTCTTGGGGATTGGTTAAAAAGATTCACAACACTCAAGAGGATTGACTAAGCATTCCGAGATAT TT
