# Supplementary material for: Transcriptional profile of genes involved in the production of terpenes and glyceollins in response to biotic stresses in soybean
Source: Genet Mol Biol. 2020 Nov 6;43(4):e20190388. doi: 10.1590/1678-4685-GMB-2019-0388 (PMC7644969; doi:10.1590/1678-4685-GMB-2019-0388)
Supplement: Supplementary file 2 [file 1415-4757-GMB-43-4-e20190388-suppl3.pdf]

Genetics and Molecular Biology
DOI: https://doi.org/10.1590/1678-4685-GMB-2019-0388

Supplementary Material to "Transcriptional profile of genes involved in the production of terpenes and glyceollins in response to biotic stresses in soybean"

Table S3 - Transcriptional characterization of mevalonate route genes in response to different soybean diseases.

Gene	Genotype	Stress	Period	Expression	log2-ratio	Fold-change	*p*-value	Author
Glyma.17g005300 E.C. 2.3.1.9 Acetyl-CoA synthase	PI200492 (Rpp1)	P. pachyrhizi (HW94-1)	6-12 hai	+	0,80	1,72	0,045	Choi *et al.*, 2008
		P. pachyrhizi (TW72-1)	6-12 hai	+	0,81	1,70	0,015	
		P. pachyrhizi (TW72-1)	48 hai	-	-1,04	-2,13	0,036	
	SD01-76R (S) e LD05-16060 (R)	Pulgões		+	1,02	2,02	0,008	Studham *et al.*, 2013
	PI459025B (Rpp4)	P. pachyrhizi (HW94-1)	12hai	-	-1,69	-3,47	0,006	Morales *et al.*, 2013
			24hai	-	-2,92	-6,73	0,013	
			48hai	+	1,12	2,14	0,001	
	SLOAN	P. sojae (PT2004C2.S1)- Raiz	24hai	+	0,41	1,33	0,001	Zhou *et al.*, 2009
	VP-RIL9	P. sojae (PT2004C2.S1)- Raiz	24hai	+	0,70	1,60	0,001	
	SLOAN	P. sojae (PT2004C2.S1)- Raiz	48hai	+	0,44	1,37	0,001	
	SLOAN	P. sojae (PT2004C2.S1)- Raiz	72hai	+	0,36	1,28	0,001	
	VP-RIL9	P. sojae (PT2004C2.S1)- Raiz	72hai	+	0,65	1,53	0,001	
	VP-RIL9	P. sojae (PT2004C2.S1)- Raiz	48hai	+	0,78	1,63	0,001	
	CONRAD	P. sojae (PT2004C2.S1)- Raiz	48hai	+	1,76	3,53	0,002	Tyler *et al.*, 2007
	SLOAN	P. sojae (PT2004C2.S1)- Raiz	48hai	+	1,64	3,38	0,005	
	V71-370	P. sojae (PT2004C2.S1)- Raiz	72hai	+	0,82	1,81	0,007	
	CONRAD	P. sojae (PT2004C2.S1)- Raiz	72hai	+	0,99	2,09	0,004	
	SLOAN	P. sojae (PT2004C2.S1)- Raiz	72hai	+	1,10	2,27	0,007	
	V71-370	P. sojae (PT2004C2.S1)- Raiz	72hai	+	0,74	1,71	0,01	
	VP-RIL9	P. sojae (PT2004C2.S1)- Raiz	72hai	+	0,67	1,59	0,004	

Gene	Genotype	Stress	Period	Expression	log2-ratio	Fold-change	*p*-value	Author
Glyma.01g215500 E.C. 2.3.3.10 Hydroxy methyl glutaryl- CoA synthase	PI200492 (Rpp1)	P. pachyrhizi (HW94-1)	48 hai	+	1,99	3,7	0,027	Choi *et al.,* 2008
	PI200492 (Rpp1)	P. pachyrhizi (TW72-1)	48 hai	+	1,63	3,09	0,04	
	PI462312	P. pachyrhizi (HW94-1)	24hai	-	-0,92	-1,88	0,029	Schneider *et al.,* 2011
	PI459025B (Rpp4)	P. pachyrhizi (HW94-1)	24hai	-	-0,77	-1,71	0,004	Morales *et al.,* 2013
	PI291327	P. sojae (PT2004C2.S1)- Raiz	72hai	+	1,18	2,39	0,008	Tyler *et al.,* 2007
	SLOAN	P. sojae (PT2004C2.S1)- Raiz	72hai	+	0,79	1,68	0,026	
	WILLIANS	P. sojae (PT2004C2.S1)- Raiz	72hai	-	-0,80	1,76	0,016	
	V71-370	P. sojae (PT2004C2.S1)- Raiz	24hai	-	-0,37	-1,29	0,049	
	CONRAD	P. sojae (PT2004C2.S1)- Raiz	48 hai	-	-1,38	-2,64	0,001	
	SLOAN	P. sojae (PT2004C2.S1)- Raiz	48 hai	-	-1,59	-3,02	0,001	
	V71-370	P. sojae (PT2004C2.S1)- Raiz	48 hai	-	-0,95	-1,89	0,018	
	VP-RIL9	P. sojae (PT2004C2.S1)- Raiz	48 hai	-	-1,25	-2,29	0,006	
	V71-370	P. sojae (PT2004C2.S1)- Raiz	72hai	-	-0,51	-1,40	0,029	
	SLOAN	P. sojae (PT2004C2.S1)- Raiz	24hai	+	1,29	2,48	0,001	Zhou *et al.,* 2009
	V71-370	P. sojae (PT2004C2.S1)- Raiz	24hai	+	1,00	1,95	0,001	
	VP-RIL9	P. sojae (PT2004C2.S1)- Raiz	24hai	-	-0,72	-1,61	0,002	
	SLOAN	P. sojae (PT2004C2.S1)- Raiz	48 hai	+	1,15	2,19	0,001	
	V71-370	P. sojae (PT2004C2.S1)- Raiz	48 hai	+	0,71	1,73	0,003	
	VP-RIL9	P. sojae (PT2004C2.S1)- Raiz	48 hai	-	-1,19	-2,14	0,001	
	SLOAN	P. sojae (PT2004C2.S1)- Raiz	72hai	+	1,44	**2,7**	**0,001**	
	V71-370	P. sojae (PT2004C2.S1)- Raiz	72hai	+	0,64	1,6	0,002	
	VP-RIL9	P. sojae (PT2004C2.S1)- Raiz	72hai	-	-0,96	-1,94	0,001	
	HAROSOY	P. sojae (strain P6497)	12hai	-	-1,86	-3,85	0,007	Gijzen *et al.,* 2009
			24hai	-	-3,86	-15,29	0,001	
			48hai	-	-6,53	-95,14	0,001	

Gene	Genotype	Stress	Period	Expression	log2-ratio	Fold-change	*p*-value	Author
Glyma.03g239000 E.C.2.7.1.36 Mevalonate kinase	PI462312	P. pachyrhizi (HW94-1)	72hai	-	-0,56	-1,48	0,019	Schneider *et al.*, 2011
	WILLIAMS	P. pachyrhizi (HW94-1)	72hai	-	-0,76	-1,74	0,036	Morales *et al.*, 2013
	OX20-8	P. sojae	72hai	+	0,48	1,39	0,009	Tyler *et al.*, 2007
	PI291327	P. sojae	72hai	+	0,35	1,28	0,024	
	SLOAN	P. sojae	72hai	+	0,59	1,51	0,001	
	V71-370	P. sojae (PT2004C2.S1)- Raiz	24hai	-	-0,38	-1,30	0,025	
	CONRAD	P. sojae (PT2004C2.S1)- Raiz	48hai	-	-0,47	-1,39	0,03	
	SLOAN	P. sojae (PT2004C2.S1)- Raiz	72hai	+	0,40	1,34	0,017	
	SLOAN	P. sojae (PT2004C2.S1)- Raiz	24hai	+	0,44	1,38	0,001	Zhou *et al.*, 2009
	V71-370	P. sojae (PT2004C2.S1)- Raiz	24hai	+	0,23	1,19	0,001	
	SLOAN	P. sojae (PT2004C2.S1)- Raiz	48 hai	+	0,51	1,45	0,001	
	V71-370	P. sojae (PT2004C2.S1)- Raiz	48 hai	+	0,22	1,18	0,001	
	SLOAN	P. sojae (PT2004C2.S1)- Raiz	72hai	+	0,51	1,43	0,001	
	V71-370	P. sojae (PT2004C2.S1)- Raiz	72hai	+	0,20	1,43	0,001	
	HAROSOY	P. sojae (strain P6497)	24hai	-	-2,03	-5,20	0,045	Gijzen *et al.*, 2009
			48hai	-	-2,74	-5,20	0,002	

Gene	Genotype	Stress	Period	Expression	log2-ratio	Fold-change	*p*-value	Author
Glyma.06g127200 E.C. 2.7.4.2 Phospho mevalonate kinase	PI462312	P. pachyrhizi (HW94-1)	12hai	-	-0,27	-1,20	0,019	Schneider *et al.*, 2011
	PI200492 (Rpp1)	P. pachyrhizi (HW94-1)	6-12hai	-	-0,42	-1,34	0,023	*Choi et al.*, 2008
	V71-370	P. sojae (PT2004C2.S1)- Raiz	24hai	-	-0,42	-1,32	0,036	Tyler *et al.*, 2007
	CONRAD	P. sojae (PT2004C2.S1)- Raiz	48 hai	-	-0,59	-1,52	0,004	
	SLOAN	P. sojae (PT2004C2.S1)- Raiz	48 hai	-	-0,71	-1,64	0,003	
	VP-RIL9	P. sojae (PT2004C2.S1)- Raiz	48 hai	-	-0,47	-1,38	0,025	
	SLOAN	P. sojae (PT2004C2.S1)- Raiz	24hai	+	0,18	1,17	0,048	Zhou *et al.*, 2009
	VP-RIL9	P. sojae (PT2004C2.S1)- Raiz	48hai	-	-0,17	-1,13	0,029	
	SLOAN	P. sojae (PT2004C2.S1)- Raiz	72hai	+	0,28	1,23	0,002	
Glyma.10g279800 E.C. 4.1.1.33 Diphospho mevalonate decarboxylase	V71-370	P. sojae	72hai	-	-0,19	-1,14	0,034	Tyler *et al.*, 2007
	CONRAD	P. sojae (PT2004C2.S1)- Raiz	48 hai	-	-0,69	-1,61	0,001	
	SLOAN	P. sojae (PT2004C2.S1)- Raiz	48 hai	-	-0,64	-1,56	0,001	
	VP-RIL9	P. sojae (PT2004C2.S1)- Raiz	48 hai	-	-0,37	-1,30	0,007	
	VP-RIL9	P. sojae (PT2004C2.S1)- Raiz	24hai	-	-0,38	-1,30	0,001	Zhou *et al.*, 2009
	VP-RIL9	P. sojae (PT2004C2.S1)- Raiz	48hai	-	-0,45	-1,37	0,001	
	VP-RIL9	P. sojae (PT2004C2.S1)- Raiz	72hai	-	-0,39	-1,31	0,001	
	HAROSOY	P. sojae (strain P6497)	48hai	-	-0,99	-2,06	0,004	Gijzen *et al.*, 2009

Gene	Genotype	Stress	Period	Expression	log2-ratio	Fold-change	*p*-value	Author
Glyma.20g109900 E.C. 4.1.1.33 Diphospho mevalonate decarboxylase	SD01-76R (susc)	pulgões	24hai	+	0,28	1,22	0,024	Studham *et al.*, 2013
	PI200492 -KOMATA (Rpp1)	P. pachyrhizi (HW94-1)	48hai	+	0,72	1,63	0,037	Choi *et al.*, 2008
	CONRAD	P. sojae	72hai	+	0,50	1,42	0,006	Tyler *et al.*, 2007
	OX20-8	P. sojae	72hai	+	0,87	1,83	0,001	
	SLOAN	P. sojae	72hai	+	1,16	2,25	0,001	
	CONRAD	P. sojae (PT2004C2.S1)- Raiz	48 hai	-	-0,93	-1,92	0,001	Tyler *et al.*, 2007
	SLOAN	P. sojae (PT2004C2.S1)- Raiz	48 hai	-	-0,88	-1,85	0,001	
	V71-370	P. sojae (PT2004C2.S1)- Raiz	48 hai	-	-0,52	-1,43	0,013	
	VP-RIL9	P. sojae (PT2004C2.S1)- Raiz	48 hai	-	-0,77	-1,7	0,011	
	SLOAN	P. sojae (PT2004C2.S1)- Raiz	72hai	+	0,77	1,83	0,036	
	SLOAN	P. sojae (PT2004C2.S1)- Raiz	24hai	+	0,85	1,83	0,001	Zhou *et al.*, 2009
	V71-370	P. sojae (PT2004C2.S1)- Raiz	24hai	+	0,74	1,72	0,001	
	VP-RIL9	P. sojae (PT2004C2.S1)- Raiz	24hai	-	-0,24	-1,21	0,033	
	SLOAN	P. sojae (PT2004C2.S1)- Raiz	48 hai	+	0,90	1,87	0,001	
	V71-370	P. sojae (PT2004C2.S1)- Raiz	48 hai	+	0,60	1,58	0,001	
	VP-RIL9	P. sojae (PT2004C2.S1)- Raiz	48 hai	-	-0,36	-1,29	0,001	
	SLOAN	P. sojae (PT2004C2.S1)- Raiz	72hai	+	1,03	2,04	0,001	
	V71-370	P. sojae (PT2004C2.S1)- Raiz	72hai	+	0,49	1,43	0,001	
	VP-RIL9	P. sojae (PT2004C2.S1)- Raiz	72hai	-	-0,22	-1,17	0,018	
	HAROSOY	P. sojae (strain P6497)	12hai	-	-1,05	-2,07	0,001	Gijzen *et al.*, 2009
			24hai	-	-2,13	-4,94	0,001	
			48hai	-	-0,18	-1,13	0,001	

Gene	Genotype	Stress	Period	Expression	log2-ratio	Fold-change	*p*-value	Author
Glyma.18g242300 E.C. 5.3.3.2 Isopentenyl diphosphate isomerase	WILLIAMS 82	H. glycines	48hai	+	1,50	2,82	0,001	Ithal *et al.,* 2007
	WILLIAMS 82	H. glycines	48hai	+	0,30	1,23	0,003	Ithal *et al.,* 2007
	PI462312	P. pachyrhizi (HW94-1)	12hai	+	4,15	18,5	0,001	Schneider *et al.,* 2011
	PI462312	P. pachyrhizi (TW80-2)	12hai	+	3,19	9,37	0,001	
	PI462312	P. pachyrhizi (TW80-2)	24hai	+	0,44	1,35	0,019	
	PI459025B (Rpp4)	P. pachyrhizi (HW94-1)	12hai	+	2,81	6,87	0,001	Morales *et al.,* 2013
	WILLIAMS	P. pachyrhizi (HW94-1)	12hai	+	2,89	6,65	0,01	
	PI459025B (Rpp4)	P. pachyrhizi (HW94-1)	24hai	+	1,89	2,26	0,008	
	WILLIAMS	P. pachyrhizi (HW94-1)	24hai	+	0,78	1,75	0,022	
	ATHOW	P. sojae	72hai	+	2,60	6,34	0,001	Tyler *et al.,* 2007
	CONRAD	P. sojae	72hai	+	3,26	9,5	0,001	
	GENERAL	P. sojae	72hai	+	2,80	7,04	0,001	
	OX20-8	P. sojae	72hai	+	3,09	8,52	0,001	
	SLOAN	P. sojae	72hai	+	3,54	11,54	0,001	
	V71-370	P. sojae	72hai	+	2,99	7,89	0,001	
	WILLIAMS	P. sojae	72hai	+	2,57	5,91	0,001	
	CONRAD	P. sojae (PT2004C2.S1)- Raiz	48 hai	+	2,17	4,56	0,001	Tyler *et al.,* 2007
	SLOAN	P. sojae (PT2004C2.S1)- Raiz	48 hai	+	1,85	3,59	0,001	
	V71-370	P. sojae (PT2004C2.S1)- Raiz	48 hai	+	1,80	3,52	0,001	
	VP-RIL9	P. sojae (PT2004C2.S1)- Raiz	48 hai	+	2,21	4,86	0,001	
	CONRAD	P. sojae (PT2004C2.S1)- Raiz	72hai	+	3,03	8,76	0,001	
	SLOAN	P. sojae (PT2004C2.S1)- Raiz	72hai	+	3,03	8,46	0,001	
	V71-370	P. sojae (PT2004C2.S1)- Raiz	72hai	+	2,32	5,41	0,001	
	VP-RIL9	P. sojae (PT2004C2.S1)- Raiz	72hai	+	2,68	6,41	0,001	

Gene	Genotype	Stress	Period	Expression	log2-ratio	Fold-change	*p*-value	Author
Glyma.18g242300 E.C. 5.3.3.2 Isopentenyl diphosphate isomerase	SLOAN	P. sojae (PT2004C2.S1)- Raiz	24hai	+	2,61	6,29	0,001	Zhou *et al.*, 2009
	V71-370	P. sojae (PT2004C2.S1)- Raiz	24hai	+	2,11	4,51	0,001	
	VP-RIL9	P. sojae (PT2004C2.S1)- Raiz	24hai	+	3,08	7,95	0,001	
	SLOAN	P. sojae (PT2004C2.S1)- Raiz	48 hai	+	2,16	4,60	0,001	
	V71-370	P. sojae (PT2004C2.S1)- Raiz	48 hai	+	1,81	3,73	0,001	
	VP-RIL9	P. sojae (PT2004C2.S1)- Raiz	48 hai	+	3,02	8,20	0,001	
	SLOAN	P. sojae (PT2004C2.S1)- Raiz	72hai	+	2,36	5,17	0,001	
	V71-370	P. sojae (PT2004C2.S1)- Raiz	72hai	+	1,78	3,26	0,001	
	VP-RIL9	P. sojae (PT2004C2.S1)- Raiz	72hai	+	3,21	8,80	0,001	
	HAROSOY	P. sojae (strain P6497)	24hai	+	0,65	1,56	0,015	Gijzen *et al.*, 2009
			48hai	-	-0,61	-1,51	0,028	
Glyma.17g166000 E.C. 2.5.1.1 Geranyl diphosphate synthase	SD01-76R (susc)	pulgões	24hai	+	0,23	1,18	0,044	Studham *et al.*, 2013
	PI462312	P. pachyrhizi (TW80-2)	12hai	+	1,39	2,62	0,001	Schneider *et al.*, 2011
	PI462312	P. pachyrhizi (HW94-1)	12hai	+	1,83	3,56	0,001	
	PI459025B (Rpp4)	P. pachyrhizi (HW94-1)	12hai	+	1,55	2,93	0,001	Morales *et al.*, 2013
	WILLIAMS	P. pachyrhizi (HW94-1)	12hai	+	1,59	2,97	0,002	
	WILLIAMS	P. pachyrhizi (HW94-1)	24hai	+	0,41	1,33	0,026	
	PI200492 (Rpp1)	P. pachyrhizi (HW94-1)	24hai	+	0,89	1,88	0,036	Choi *et al.*, 2008
	PI200492 (Rpp1)	P. pachyrhizi (HW94-1)	48 hai	+	1,34	2,53	0,006	
	PI200492 (Rpp1)	P. pachyrhizi (TW72-1)	48 hai	+	0,88	1,87	0,02	

Gene	Genotype	Stress	Period	Expression	log2-ratio	Fold-change	*p*-value	Author
	ATHOW	P. sojae	72hai	+	0,97	1,98	0,001	Tyler *et al.,* 2007
	CONRAD	P. sojae	72hai	+	0,98	1,96	0,001	
	GENERAL	P. sojae	72hai	+	1,09	2,12	0,001	
	OX20-8	P. sojae	72hai	+	1,39	2,63	0,001	
	PI291327	P. sojae	72hai	+	1,25	2,37	0,001	
	SLOAN	P. sojae	72hai	+	1,31	2,47	0,001	
	V71-370	P. sojae	72hai	+	0,97	1,97	0,002	
	WILLIAMS	P. sojae	72hai	+	1,18	2,27	0,001	
	CONRAD	P. sojae (PT2004C2.S1)- Raiz	48 hai	+	0,95	1,93	0,003	Tyler *et al.,* 2007
	SLOAN	P. sojae (PT2004C2.S1)- Raiz	48 hai	+	0,95	1,94	0,001	
	V71-370	P. sojae (PT2004C2.S1)- Raiz	48 hai	+	0,74	1,67	0,001	
	VP-RIL9	P. sojae (PT2004C2.S1)- Raiz	48 hai	+	0,90	1,93	0,014	
	CONRAD	P. sojae (PT2004C2.S1)- Raiz	72hai	+	1,16	2,25	0,001	
	SLOAN	P. sojae (PT2004C2.S1)- Raiz	72hai	+	1,16	2,27	0,001	
	V71-370	P. sojae (PT2004C2.S1)- Raiz	72hai	+	0,75	1,69	0,001	
	VP-RIL9	P. sojae (PT2004C2.S1)- Raiz	72hai	+	0,95	1,97	0,001	
Glyma.17g166000 E.C. 2.5.1.1 Geranyl diphosphate synthase	SLOAN	P. sojae (PT2004C2.S1)- Raiz	24hai	+	0,54	1,49	0,001	Zhou *et al.,* 2009
	V71-370	P. sojae (PT2004C2.S1)- Raiz	24hai	+	0,47	1,39	0,001	
	VP-RIL9	P. sojae (PT2004C2.S1)- Raiz	24hai	+	0,84	1,81	0,001	
	SLOAN	P. sojae (PT2004C2.S1)- Raiz	48 hai	+	0,43	1,37	0,001	
	V71-370	P. sojae (PT2004C2.S1)- Raiz	48 hai	+	0,49	1,41	0,001	
	VP-RIL9	P. sojae (PT2004C2.S1)- Raiz	48 hai	+	0,93	1,92	0,001	
	SLOAN	P. sojae (PT2004C2.S1)- Raiz	72hai	+	0,56	1,48	0,001	
	V71-370	P. sojae (PT2004C2.S1)- Raiz	72hai	+	0,42	1,36	0,001	
	VP-RIL9	P. sojae (PT2004C2.S1)- Raiz	72hai	+	1,05	2,10	0,001	
	HAROSOY	P. sojae (strain P6497)	48hai	-	-1,16	-2,32	0,018	Gijzen *et al.,* 2009

Gene	Genotype	Stress	Period	Expression	log2-ratio	Fold-change	p-value	Author
Glyma.15g121400<br>E.C.2.5.1.10<br>(2E, 6E)- farnesyl diphosphate synthase	SD01-76R (susc)	pulgões	24hai	+	0,56	1,46	0,023	Studham *et al.,* 2013
	PI200492 (Rpp1)	P. pachyrhizi (HW94-1)	6-12 hai	-	-1,04	-2,09	0,018	Choi *et al.,* 2008
	PI200492 (Rpp1)	P. pachyrhizi (TW72-1)	6-12 hai	-	-1,12	-2,24	0,008	
	WILLIAMS	P. sojae	72hai	-	-0,72	-1,66	0,045	Tyler *et al.,* 2007
	SLOAN	P. sojae (PT2004C2.S1)- Raiz	24hai	-	-0,32	-1,24	0,042	Tyler *et al.,* 2007
	CONRAD	P. sojae (PT2004C2.S1)- Raiz	48 hai	-	-1,04	-2,09	0,031	
	SLOAN	P. sojae (PT2004C2.S1)- Raiz	48 hai	-	-1,13	-2,12	0,006	
	V71-370	P. sojae (PT2004C2.S1)- Raiz	48 hai	-	-0,96	-1,89	0,011	
	VP-RIL9	P. sojae (PT2004C2.S1)- Raiz	48 hai	-	-1,24	-2,27	0,020	
	SLOAN	P. sojae (PT2004C2.S1)- Raiz	24hai	+	0,26	1,17	0,047	Zhou *et al.,* 2009
	V71-370	P. sojae (PT2004C2.S1)- Raiz	24hai	+	0,4	1,29	0,001	
	VP-RIL9	P. sojae (PT2004C2.S1)- Raiz	24hai	-	-0,56	-1,41	0,006	
	V71-370	P. sojae (PT2004C2.S1)- Raiz	48 hai	+	0,36	1,32	0,026	
	VP-RIL9	P. sojae (PT2004C2.S1)- Raiz	48 hai	-	-0,6	-1,49	0,001	
	SLOAN	P. sojae (PT2004C2.S1)- Raiz	72hai	+	0,38	1,27	0,001	
	VP-RIL9	P. sojae (PT2004C2.S1)- Raiz	72hai	-	-0,59	-1,55	0,001	
	HAROSOY	P. sojae (strain P6497)	12hai	-	-1,2	-2,4	0,019	Gijzen *et al.,* 2009
			24hai	-	-2,48	-5,49	0,007	
			48hai	-	-5,52	-47,52	0,001	

Gene	Genotype	Stress	Period	Expression	log2-ratio	Fold-change	p-value	Author
Glyma.11g063900 E.C. 2.5.1.29 Geranyl-geranyl diphosphate synthase	LD05-16060	Pulgões	24hai	+	0,34	1,27	0,014	Studham *et al.,* 2013
	PI200492 (Rpp1)	P. pachyrhizi (HW94-1)	6-12 hai	-	-1,34	-2,62	0,03	Choi *et al.,* 2008
	PI200492 (Rpp1)	P. pachyrhizi (TW72-1)	6-12 hai	-	-1,06	-2,18	0,048	
	CONRAD	P. sojae	72hai	+	0,38	1,3	0,001	Tyler *et al.,* 2007
	GENERAL	P. sojae	72hai	+	0,34	1,25	0,04	
	PI291327	P. sojae	72hai	+	0,41	1,34	0,014	
	SLOAN	P. sojae	72hai	+	0,83	1,76	0,001	
	WILLIAMS	P. sojae	72hai	+	0,48	1,4	0,002	
	SLOAN	P. sojae (PT2004C2.S1)- Raiz	48 hai	-	-0,34	-1,25	0,029	Tyler *et al.,* 2007
	CONRAD	P. sojae (PT2004C2.S1)- Raiz	72hai	+	0,35	1,3	0,032	
	SLOAN	P. sojae (PT2004C2.S1)- Raiz	72hai	+	0,39	1,32	0,02	
	V71-370	P. sojae (PT2004C2.S1)- Raiz	72hai	+	0,31	1,25	0,045	
	SLOAN	P. sojae (PT2004C2.S1)- Raiz	24hai	+	0,47	1,38	0,001	Zhou *et al.,* 2009
	V71-370	P. sojae (PT2004C2.S1)- Raiz	24hai	+	0,39	1,31	0,001	
	SLOAN	P. sojae (PT2004C2.S1)- Raiz	48 hai	+	0,46	1,39	0,001	
	V71-370	P. sojae (PT2004C2.S1)- Raiz	48 hai	+	0,19	1,15	0,003	
	SLOAN	P. sojae (PT2004C2.S1)- Raiz	72hai	+	0,47	1,4	0,001	
	V71-370	P. sojae (PT2004C2.S1)- Raiz	72hai	+	0,18	1,14	0,001	
	HAROSOY	P. sojae (strain P6497)	48hai	-	-0,85	-1,81	0,002	Gijzen *et al.,* 2009

Gene	Genotype	Stress	Period	Expression	log2-ratio	Fold-change	p-value	Author
Glyma.10g295300 E.C. 2.5.1.36 Glyceollin synthase	WILLIAMS 82	B. japonicum	6hai	+	0,75	1,66	0,006	Libaut *et al.,* 2010
	PI462312	P. pachyrhizi (HW94-1)	24hai	+	0,17	1,12	0,034	Schneider *et al.,* 2011
	PI462312	P. pachyrhizi (TW80-2)	24hai	+	0,29	1,22	0,001	
	WILLIAMS	P. pachyrhizi (HW94-1)	12hai	+	0,14	1,10	0,015	Morales *et al.,* 2013
	PI200492 (Rpp1)	P. pachyrhizi (TW72-1)	48hai	+	0,39	1,31	0,02	Choi *et al.,* 2008
	PI200492 (Rpp1)	P. pachyrhizi (TW72-1)	96hai	-	-0,19	-1,14	0,001	
	OX20-8	P. sojae	72hai	-	-0,28	-1,21	0,042	Tyler *et al.,* 2007
	SLOAN	P. sojae	72hai	+	0,25	1,19	0,009	
	CONRAD	P. sojae (PT2004C2.S1)- Raiz	24hai	-	-0,42	-1,32	0,025	Tyler *et al.,* 2007
	CONRAD	P. sojae (PT2004C2.S1)- Raiz	48 hai	-	-1,59	-2,99	0,001	
	SLOAN	P. sojae (PT2004C2.S1)- Raiz	48 hai	-	-1,41	-2,54	0,002	
	V71-370	P. sojae (PT2004C2.S1)- Raiz	48 hai	-	-0,83	-1,70	0,032	
	VP-RIL9	P. sojae (PT2004C2.S1)- Raiz	48 hai	-	-1,04	-2,02	0,01	
	CONRAD	P. sojae (PT2004C2.S1)- Raiz	72hai	-	-0,50	-1,39	0,015	
	V71-370	P. sojae (PT2004C2.S1)- Raiz	72hai	-	-0,43	-1,36	0,003	
	SLOAN	P. sojae (PT2004C2.S1)- Raiz	24hai	+	0,69	1,66	0,001	Zhou *et al.,* 2009
	V71-370	P. sojae (PT2004C2.S1)- Raiz	24hai	+	0,50	1,41	0,001	
	SLOAN	P. sojae (PT2004C2.S1)- Raiz	48 hai	+	0,57	1,59	0,001	
	SLOAN	P. sojae (PT2004C2.S1)- Raiz	72hai	+	0,72	1,69	0,001	
	HAROSOY	P. sojae (strain P6497)	48hai	+	0,51	1,41	0,017	Gijzen *et al.,* 2009

Gene	Genotype	Stress	Period	Expression	log2-ratio	Fold-change	p-value	Author
Glyma.13g321100 E.C. 4.2.3.46 α-farnesene synthase	PI459025B (Rpp4)	P. pachyrhizi (HW94-1)	72hai	+	1,71	3,20	0,008	Morales *et al.*, 2013
	WILLIAMS	P. pachyrhizi (HW94-1)	>200hai	-	-4,61	-22,4	0,004	
	PI200492 (Rpp1)	P. pachyrhizi (HW94-1)	6-12 hai	-	-1,71	-3,51	0,004	Choi *et al.*, 2008
	PI200492 (Rpp1)	P. pachyrhizi (TW72-1)	6-12 hai	-	-1,86	-3,99	0,001	
	PI200492 (Rpp1)	P. pachyrhizi (HW94-1)	48 hai	+	5,01	34,66	0,011	
	PI200492 (Rpp1)	P. pachyrhizi (TW72-1)	48 hai	+	3,28	12,55	0,038	
	V71-370	P. sojae (PT2004C2.S1)- Raiz	24hai	-	-0,08	-1,06	0,041	Zhou *et al.*, 2009
	VP-RIL9	P. sojae (PT2004C2.S1)- Raiz	24hai	-	-0,18	-1,13	0,001	
	SLOAN	P. sojae (PT2004C2.S1)- Raiz	48 hai	-	-0,1	-1,07	0,038	
	VP-RIL9	P. sojae (PT2004C2.S1)- Raiz	48 hai	-	-0,18	-1,13	0,001	
	SLOAN	P. sojae (PT2004C2.S1)- Raiz	72hai	-	-0,12	-1,09	0,005	
	VP-RIL9	P. sojae (PT2004C2.S1)- Raiz	72hai	-	-0,17	-1,12	0,001	

Genevestigator: Essay formed for soybena plants (V3) in three treatments: with virulent strain of P. pachyrhizi (TW80-2), with avirulent strains (HW94-1) and controls (simulated), with 6 points: 12hpi 24hpi, 72hpi, 144hpi, 216hpi, 288hpi. Experiment data available http://www.plexdb.org/modules/PD_browse/experiment_browser.php?experiment=GM36. All other data were obtained by consulting the Genevestigator (https://www.genevestigator.com/gv/plant.jsp), using the parameters for an analysis of the results: p<0,05; ratio≥0,5; fold-change ≥1,0. Gene IDs accompanied for "*"represent 69 models induction between soybean-FAS; – *P.sojae; H. glycines; S. litura; B. japonicum. Aphis glycine*s x soja (V3): Transcriptional analysis response of soybean plants to infestation of resistant and susceptible soybean aphids Experiment with three factors: soybean variety in V3 (susceptible SD01-76R, resistant LD05-16060), treatment of aphids (control, aphids), and duration of infestation (1 day and 7 days). Data available at: http://www.ncbi.nlm.nih.gov/geo/query/acc.cgi?acc=GSE35427. Heterodera glycines: analysis soybean root transcription profiles (Williams 82), by microarray, at three timepoints of infection (2.5,10 days after inoculation - dpi) with cystic nematodes of soybean. Data available at: http://www.ebi.ac.uk/arrayexpress/browse.html?keywords=%09+E-MEXP-808. *Phytophthora sojae*: Identification of genes differentially expressed during infection by P. sojae pathogen in 8 soybean cultivars that differ in quantitative resistance (Athow,Conrad, General, Ox20-8, PI291327, Sloan, V71-370, Williams), by microarray. Analyzes were performed at times: 3dpi and 5dpi. Data available at: http://www.ncbi.nlm.nih.gov/geo/query/acc.cgi?acc=GSE7124; During the P.sojae infection (isolate PT2004C2.S1), soybean cultivars differing in quantitative resistance (resistant to strain - CONRAD e V71-370, suscetíveis – SLOAN e VP-RIL9) were observed at different times: studies (3) (24hpi), (4) (48hpi), (5) (72hpi), (6) (120hpi), available at: http://www.plexdb.org/modules/PD_browse/experiment_browser.php?experiment=GM3 e estudos (7)(24hpi),(8) (48hpi),(9) (72hpi), available at: http://www.ncbi.nlm.nih.gov/geo/query/acc.cgi?acc=GSE11611. Study 10 - Harosoy soybean hypocotyl samples (susceptible) inoculated with P. sojae strain P6497 (race 2), were analyzed by microarray at 12, 24, 48 h after inoculation (hpi). All results were obtained from microarray assays.

References

Choi JJ, Alkarouf NW, Schneider KT, Matthews BF and Frederick RD (2008) Expression patterns in soybean resistant to *Phakopsora pachyrhizi* reveal the importance of peroxidases and lipoxygenases. Funct Integr Genomics 8:341-359.

Gijzen, Dong S, Qutob D, Tedman-Jones J and Kuflu K (2009) The *Phytophthora sojae* avirulence *locus* Avr3c encodes a multi-copy RXLR effector with sequence polymorphisms among pathogen strains. PLoS One 4:5556.

Ithal N, Recknor J, Nettleton D, Hearne L, Maier T, Baum TJ and Mitchum MG (2007). Parallel genome-wide expression profiling of host and pathogen during soybean cyst nematode infection of soybean. Mol Plant Microbe Interact 20:293-305.

Libault M, Farmer A, Brechenmacher L and Drnevich J (2010) Complete transcriptome of the soybean root hair cell, a single-cell model, and its alteration in response to *Bradyrhizobium japonicum* infection. Plant Physiol 152:541-52.

Morales AMAP, O' Rourke JA, van de Mortel, Scheider KT, Bancroft TJ, Borém A, Nelson RT, Nettleton D, Baum TJ, Shoemaker RC *et al.* (2013) Transcriptome analyses and virus induced gene silencing identify genes in the *Rpp*4-mediated Asian soybean rust resistance pathway. Funct Plant Biol 40:1029-1047.

Schneider KT, van de Mortel M, Bancroft TJ, Braun E, Nettleton D, Nelson RT, Frederick RD, Baum TJ, Graham MA and Whitham SA (2011) Biphasic gene expression changes elicited by *Phakopsora pachyrhizi* in soybean correlates with fungal penetration and haustoria formation. Plant Physiol 157:355-371.

Studham ME and Macintosh GC (2013) Multiple phytohormone signals control the transcriptional response to soybean aphid infestation in susceptible and resistant soybean plants. Mol Plant Microbe Interact 26:116-129.

Tyler BM (2007) *Phytophthora sojae*: Root rot pathogen of soybean and model *oomycete*. Mol Plant Pathol 8:1-8.

Zhou L, Mideros SX, Bao L and Hanlon R (2009) Infection and genotype remodel the entire soybean transcriptome. BMC Genomics 26:10-49.
