# Supplementary material for: Transcriptional profile of genes involved in the production of terpenes and glyceollins in response to biotic stresses in soybean
Source: Genet Mol Biol. 2020 Nov 6;43(4):e20190388. doi: 10.1590/1678-4685-GMB-2019-0388 (PMC7644969; doi:10.1590/1678-4685-GMB-2019-0388)
Supplement: Supplementary file 3 [file 1415-4757-GMB-43-4-e20190388-suppl2.pdf]

Genetics and Molecular Biology
DOI: https://doi.org/10.1590/1678-4685-GMB-2019-0388

Supplementary Material to "Transcriptional profile of genes involved in the production of terpenes and glyceollins in response to biotic stresses in soybean"

Table S2 - Expression profile of genes involved in Terpenoid and Glyceollin biosynthesis in soybean after *P. pachyrhizi* infection.

ID phytozome V.10	Genotype	12 hai	24 hai	48 hai	72 hai	96 hai	192 hai
Glyma17g005300 E.C. 2.3.1.9 Acetyl-CoA acetyltransferase	W82	0,867	0,865	1,601*	1,061	0,818*	1,632*
	Rpp2	0,803*	1,205	1,28*	1,138	0,729	1,497*
	Rpp4	1,331	1,225	1,035	0,884*	0,938	
	Rpp5	1,068	1,575	9,627	1,401*	0,997	2,225*
Glyma01g215500 E.C 2.3.3.10 Hydroxymethylglutaryl-CoA synthase	W82	0,97	0,779	1,32*	0,487*	1,002	0,45*
	Rpp2	0,71*	0,482*	0,776	0,458*	0,495*	1,405
	Rpp4	0,953	0,477*	1,667*	1,199	0,602*	
	Rpp5	1,116	0,009*	0,165*	1,159	0,772	1,271
Glyma03g239000 E.C. 2.7.1.36 Mevalonate kinase	W82	1,158	0,619*	0,668	0,35*	0,7	1,329*
	Rpp2	0,559*	1,046	0,658	0,872	0,709*	0,547*
	Rpp4	0,657*	1,959	1,244	1,468*	1,002	
	Rpp5	0,70*	7,749	1,975	1,248	1,091	0,926
Glyma06g127200 E.C. 2.7.4.2 Phosphomevalonate kinase	W82	0,722*	0,574*	0,906	0,345*	0,751	0,936
	Rpp2	0,546*	1,182	0,07*	0,963	0,555*	1,84*
	Rpp4	0,761	0,849	1,127	1,07	0,972	
	Rpp5	1,04	5,418	79,77*	2,137*	1,369*	1,113
Glyma20g109900 E.C. 4.1.133 Diphosphomevalonate decarboxylase	W82	0,954	2,201	1,173*	0,452	0,922	0,174*
	Rpp2	1,085	1,115	0,71	0,865	1,116	1,185
	Rpp4	1,442	0,731	0,997	1,236	0,209*	
	Rpp5	2,36*	6,786*	4,062*	0,977	1,206	0,809*
Glyma10g279800 E.C. 4.1.133 Diphosphomevalonate decarboxylase	W82	1,106	3,098	0,99	0,546	0,976	1,344*
	Rpp2	0,735*	1,1	0,687	1,054	0,811	1,087
	Rpp4	1,378*	0,745	0,945	1,009	0,689*	
	Rpp5	1,014	11,057*	137,027*	1,279*	1,25	0,841

ID phytozome V.10	Genotype	12 hai	24 hai	48 hai	72 hai	96 hai	192 hai
Glyma18g242300 E.C. 5.3.3.2 Isopentenyl diphosphate Δ-isomerase	W82	1,942*	0,861	1,358	0,764	1,051	1,566*
	Rpp2	1,67*	1,139	0,992	1,027	1,175	1,508*
	Rpp4	1,717*	1,132	1,284	1,37*	0,028*	
	Rpp5	1,64*	0,185*	0,078*	0,53*	0,676*	1,713*
Glyma17g166000 E.C. 2.5.1.1 Geranyl diphosphate synthase	W82	1,465*	1,071	1,21	0,7*	0,917	0,042
	Rpp2	1,026*	0,833	0,814*	0,898	1,058	1,149
	Rpp4	1,345*	0,948	1,168	1,312	1,12	
	Rpp5	1,142	1,306*	1,294*	1,195	1,233	2,096*
Glyma09g015600 E.C. 2.5.1.10 (2E,6E)- farnesyl diphosphate synthase	W82	1,26*	1,833	1,122	1,038	1,287*	0,834
	Rpp2	0,733*	0,751	0,904	0,967	0,884	0,929
	Rpp4	1,015	0,853	1,361	1,328	0,914	
	Rpp5	0,758	0,6*	1,94*	1,68*	1,478	1,29*
Glyma15g121400 E.C. 2.5.1.10 (2E,6E)- farnesyl diphosphate synthase	W82	1,197	1,112	1,054	1,262*	1,497*	0,876
	Rpp2	0,694*	0,538*	0,579*	0,791*	0,815*	0,812*
	Rpp4	0,855	0,684*	1,316*	1,328*	0,84	
	Rpp5	0,929	0,48*	0,603	1,151	1,332	2,294*
Glyma11g063900 E.C. 2.5.1.29 Geranyl-geranyl diphosphate synthase	W82	0,898	2,723	1,402*	0,411	0,627*	0,835
	Rpp2	0,973	1,351*	1,064	1,211	0,932	1,115
	Rpp4	1,006	0,366*	0,695*	0,827	0,52*	
	Rpp5	0,981	1,061	0,6*	3,403*	0,849*	0,909
Glyma13g321100 E.C 4.2.3.46 α- farnesene synthase	W82	0,231*	0,999	1,05	0,302*	0,233*	0,39*
	Rpp2	0,389*	1,165	1,336	0,342*	0,337*	0,951
	Rpp4	0,332*	0,719*	4,76*	0,39*	0,598	
	Rpp5	1,407	0,502*	0,966	0,116	0,133*	1,559
Glyma10g295300 E.C 2.5.1.36 Glyceollin synthase	W82	11,957*	1,194	5,158*	1,797	3,379*	11,72*
	Rpp2	12,758*	2,316*	4,23*	5,854*	2,009*	12,433*
	Rpp4	15,659*	1,593	1,753	0,685	4,352*	
	Rpp5	22,899*	2,349*	0,948	0,915	33,969*	21,426*

Expression values are presented in base log 2 of the RQ (relative quantification) values determined by Software REST (*Relative Expression Software Tool*) (PFAFFL *et al.*, 2009). Legend: W82 refers to susceptible cultivar Williams 82, Rpp2 - PI 230970, Rpp4 - PI459025 e Rpp5 - PI200487
* Significant values of expression at 5% probability level.

Reference

Pfaffl MW, Horgan GW and Dempfle L (2009) Relative expression software tool (REST) for group-wise comparison and statistical analysis of relative expression results in real-time PCR. Nucleic Acids Res 30:e36.
